# Immunotherapy for Cancer: Current Advances and Future Directions

**DOI:** 10.3390/ijms27167216

**Published:** 2026-08-13

**Authors:** Tanya Saxena, Shamsuz Zaman, Himanshu Dhanda, Sandeep Kumar Swain, Neetu Kushwaha, Manpreet Kaur, Raj Kamal, Sufian Zaheer, Pranay Tanwar, Neeraj Kumar, Fouzia Siraj, Bhavika Rishi, Aroonima Misra

**Affiliations:** 1 National Institute of Child Health Development Research, Indian Council of Medical Research, New Delhi 110029, India; 2 Department of Pathology, Vardhman Mahavir Medical College and Safdarjung Hospital, New Delhi 110029, India; 3 Laboratory Oncology Unit, Dr. B.R. Ambedkar Institute Rotary Cancer Hospital, AIIMS, New Delhi 110029, India; 4 Centre for Cancer Pathology, Indian Council of Medical Research, New Delhi 110029, India

**Keywords:** immunotherapy, immune checkpoint inhibitors, adoptive cell therapies, cancer vaccines, tumor microenvironment reprogramming, monoclonal antibodies, cytokine therapies, oncolytic virus therapy

## Abstract

Immunotherapy delivers a fundamental shift in the treatment of multiple cancers, offering hope for patients with cancer. Unlike conventional therapies, it leverages the patient’s immune system to precisely identify and destroy cancer cells. The remarkable journey of immunotherapy’s has revolutionized oncology and paved the way for the development of modern immunotherapies. Several approaches are applied for immunotherapy, including immune checkpoint inhibitors, adoptive cell therapies, cancer vaccines, tumor microenvironment reprogramming, monoclonal antibodies, cytokine therapies, oncolytic virus therapy and combination strategies. Various immunotherapies are being used for different types of cancers like skin, lung, bladder, kidney, liver, breast and blood cancers. Despite providing an effective cancer treatment, immunotherapies face some challenges in their clinical implementation. To overcome these limitations, future directions emphasize the development of personalized and combination therapies, the incorporation of AI and a good delivery system. The main aim of this review is to give an overview of the journey of evolution, different approaches, limitations, advances and future development for immunotherapy in cancer treatment.

## 1. Introduction

Cancer remains a prominent cause of death across the world. The estimations from the International Agency for Research on Cancer (IARC) reported that in 2022, there were 20 million new cancer cases and 9.7 million deaths [1]. There are numerous genetic and environmental factors responsible for the development of cancer. Significant advancements have been made in the understanding of causes, pathophysiology and treatment of cancer. Over the past few decades, immunotherapy has developed as an innovative treatment approach for multiple cancers. This review summarizes the different approaches for immunotherapy in cancer with a brief overview of the advances and future research associated with them.

### 1.1. Cancer and the Immune System: An Overview

The immune system helps our body fight against infections and plays a crucial role in the regulation of cancer growth and metastasis. The available literature strongly suggests that the immune system detects and controls the development of the tumor through a process called immunosurveillance [2]. The progression of cancer is controlled by both innate and adaptive immune systems. The innate immunity does not involve the specific tumor antigen recognition and provides a quick response, while adaptive immunity targets a specific tumor-associated antigen. The immune system employs different cells against different types of cancer. While T cells directly destroy the cancerous cells, natural killer cells primarily eliminate cells with absent or reduced MHC class 1 expression, and B cells produce antibodies that target specific antigens in the cancer cells. However, immunotherapies may enhance cancer treatment by empowering the immune system to fight cancer and improve the prognosis for many patients.

### 1.2. The Evolution of Cancer Treatment Approaches

Cancer treatment approaches have evolved remarkably over the past centuries [3]. Treatment methods have evolved from invasive surgical procedures to highly sophisticated therapeutic strategies. In the 19th century, with the advent of anesthesia and antiseptics, the surgical removal of tumors became the earliest form of cancer treatment. Over time, surgical techniques have become advanced and safer. After the discovery of X-rays in 1895 by Roentgen, the first non-surgical treatment option, i.e., radiation therapy, was developed [4]. High radiation damages the cancer cells’ DNA, impacting their cell division. The 20th century marked an explosion in cancer research with the introduction of systemic therapy, including chemotherapy. The discovery during World War II that nitrogen mustard could shrink lymphoma led to the development of chemotherapy as treatment of cancer [5]. For better clinical outcomes, chemotherapy is often given along with other therapies such as surgery or radiation therapy. Two or more drugs in combination can also prolong the survival rate of patients and minimize drug resistance mechanisms [6]. With the advancements in therapeutics, immunotherapy has emerged as a potential approach for cancer treatment. It harnesses the body’s own immune system to fight against cancer and can also be given in combination with other therapies to enhance the cure rate.

### 1.3. Rationale for Immunotherapy in Cancer Management

Immunotherapy represents a major breakthrough in cancer treatment by utilizing the body’s own immune system to detect and fight cancer. Immunotherapeutic treatment has significantly improved outcomes across diverse cancer types, including breast, lung, colorectal and prostate. This therapeutic approach identifies target-specific antigens, offering high specificity with fewer side effects. Stimulating the memory of the adaptive immune system, it leads to a durable and long-lasting response for cancer treatment. Moreover, it can work synergistically with conventional therapies like radiation or chemotherapy for enhanced outcomes. Consequently, immunotherapy can be a promising approach for enhanced outcomes for cancer treatment with potential benefits over traditional methods.

## 2. History of Immunotherapy

The history of immunotherapy in cancer treatment is a fascinating journey that has led to the development of an advanced therapeutic approach. Here is an overview of this journey:

### 2.1. Early Immunotherapy Concepts

The first effective immunotherapy was developed in 1891 by William B. Coley, known as the “Father of Immunotherapy” [7]. He observed that bacterial infections sometimes resulted in tumor regression and used a mixture of live and inactivated bacteria, Streptococcus pyogenes and Serratia marcescens, often known as Coley’s toxins, to boost an immune response against tumors. However, at that time, this mechanism of immunostimulation was not understood, leading to a lack of interest in immunotherapy for several decades. In the 1950s, Lewis Thomas and Frank Macfarlane Burnet proposed the “immune surveillance theory” [8]. According to this theory, the immune system continuously searches for and destroys emerging tumor cells. When the cells evade immune surveillance, it may lead to tumor progression. In 1976, Bacillus Calmette–Guérin (BCG), the tuberculosis vaccine, was found effective in treating superficial bladder cancer [9]. Researchers suggested in the 1970s that cancer cells express tumor-specific antigens (TSAs) that distinguish them from normal cells. The early adoptive cell transfer concept, in which immune cells from patients with cancer were removed, modified outside and reinfused, was the precursor to modern CAR-T cell therapy. In the 1980s, the concept of cytokine therapy was introduced, in which Interleukin-2 and interferons were used to boost immune activity. These early concepts laid a foundation for the use of the immune system against cancer and led to the development of modern immunotherapy approaches.

### 2.2. Key Historical Milestones

Some of the key milestones in the history of immunotherapy for cancer include the early observation that bacterial infections could lead to tumor regression, the development of cytokine-based therapy, such as interferons and interleukins, for immune stimulation and the identification and FDA approval of interleukin 2 (IL-2) in 1990’s as the first immunotherapy for metastasis melanoma and renal cell carcinoma [8]. The development of monoclonal antibodies involves a single type of antibody targeting a specific antigen. The FDA authorized Rituximab as the first monoclonal antibody in 1997 to treat non-Hodgkin’s lymphoma [10]. In terms of survival and quality of life, the discovery of treatments such as checkpoint inhibitors (CPIs), chimeric antigen receptor T (CAR-T) cell therapy, cancer vaccines, and combination strategies has significantly improved cancer treatment.

### 2.3. Breakthrough Discoveries Leading to Modern Immunotherapy

**Figure 1:**
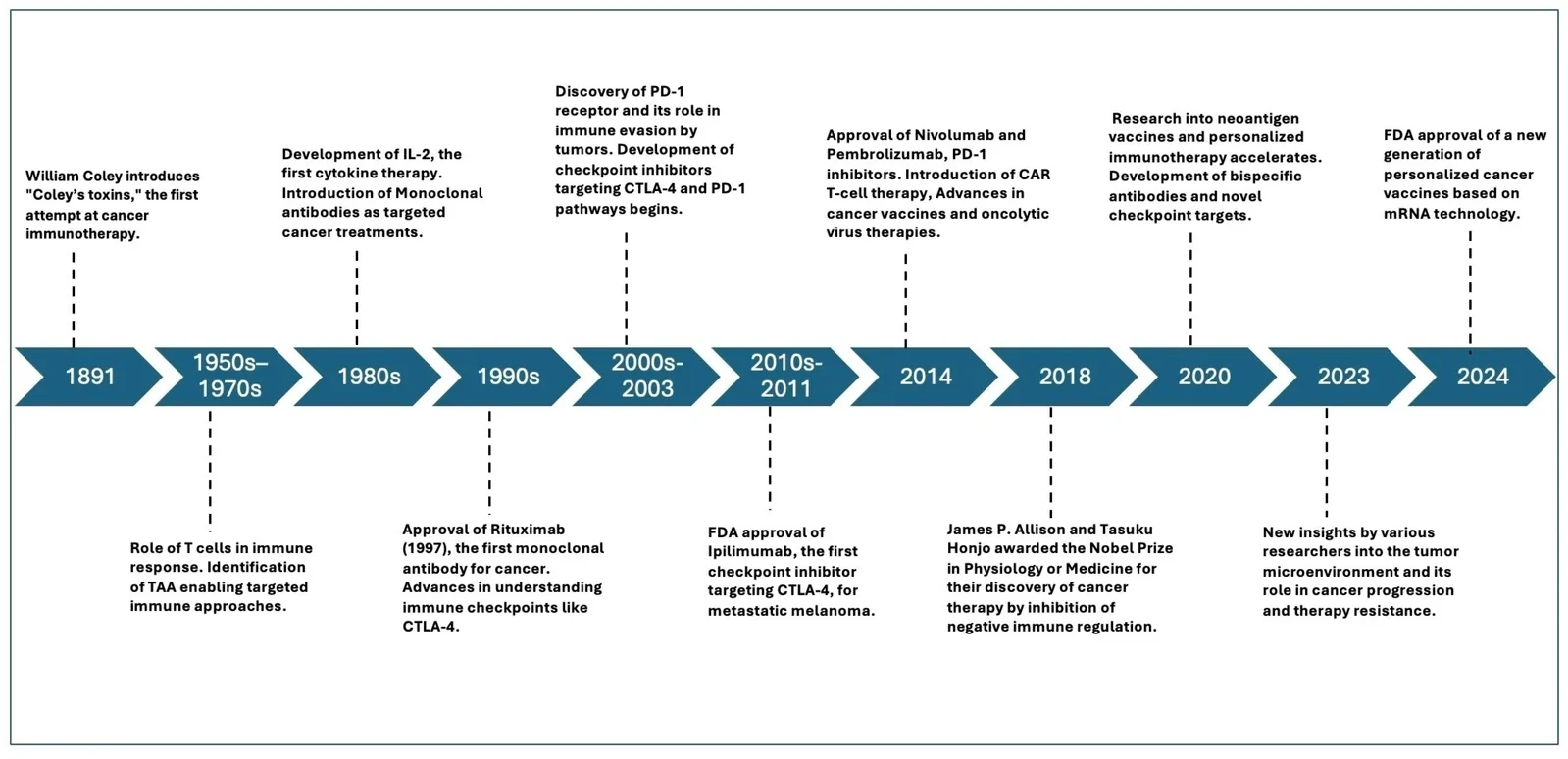
Timeline highlighting key milestones in cancer immunotherapy.

Several breakthrough discoveries in early approaches to immunotherapy have led to the development of modern immunotherapy (Figure 1) and revolutionized the field of oncology. The pivotal shift from crude immune stimulation to sophisticated, targeted approaches has significantly improved the clinical applications for various cancers. The groundbreaking breakthrough was the discovery of immune checkpoints like cytotoxic T-lymphocyte-associated protein 4 (CTLA-4) and programmed cell death protein 1 (PD-1) in the 1990s [11]. In 2011, ipilimumab (anti-CTLA-4) became the first immune checkpoint inhibitor approved by the FDA for the treatment of metastatic melanoma [12]. This was followed by the approval of nivolumab in 2014, which is an immune checkpoint point PD-1/PD-L1 inhibitor. CAR-T cell therapy has emerged as a promising strategy for cancer immunotherapy, involving the modification of a patient’s T cells to target cancer-specific antigens [13]. Research on cancer immunotherapy continues to evolve with the development of cancer vaccines and combination therapy, which has transformed the landscape of cancer immunotherapy.

## 3. Classification of Cancer Immunotherapies

**Figure 2:**
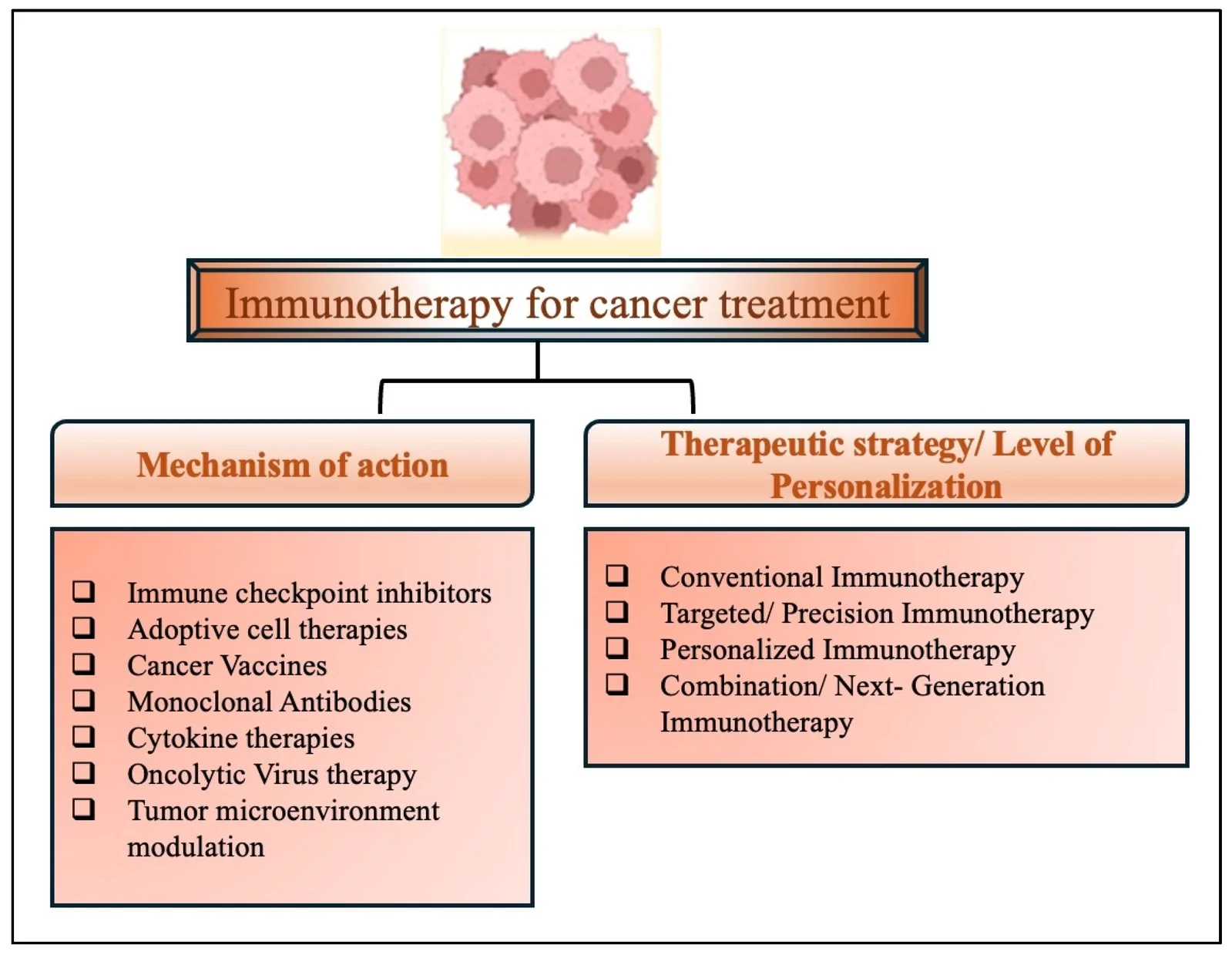
Schematic overview of classification of immunotherapy. Each approach to cancer immunotherapy has different strategies for cancer treatment.

Immunotherapy in cancer can be classified comprehensively (Figure 2) as follows:

*A.*  *Based on mechanism of action (with Predominant Site of Action):*
  1. Immune Checkpoint Inhibitors (Primarily Extracellular)—function by releasing inhibitory brakes on immune cells to strengthen anti-tumor response such as anti-PD-1, anti-PD-L1, and anti-CTLA-4 antibodies.
  2. Adoptive Cell Therapies (Both Extracellular and Intracellular)—Involve the transfer of activated or genetically engineered immune cells to the patient. Types include—CAR-T cells, TCR-T cells, TILs, and NK-cell therapies.
  3. Cancer Vaccines (Primarily Intracellular)—designed to induce or enhance immune recognition of tumor antigens, e.g., preventive vaccines, therapeutic vaccines, mRNA-based platforms, and neoantigen-based personalized vaccines (Table 1).
  4. Monoclonal Antibodies (Primarily Extracellular)—Target specific tumor antigens or immune modulators. Variants include naked antibodies, antibody-drug conjugates (ADCs), and bispecific or multispecific antibodies.
  5. Cytokine Therapies (Primarily Extracellular with Downstream Intracellular Effects)—use cytokines to stimulate immune cell proliferation and activation, e.g., IL2, IL-15, interferons, and engineered cytokines with improved properties.
  6. Oncolytic Virus Therapy (Extracellular Entry with Intracellular Action)—employs viral vectors that selectively infect and lyse tumor cells, simultaneously enhancing immune activation.
  7. Tumor Microenvironment Modulation (Both Extracellular and Intracellular)—TME modulation represents a distinct immunotherapeutic class defined by its focus on reshaping the tumor milieu. It targets suppressive immune cells (e.g., Tregs, MDSCs), remodels stromal components, or reprograms tumor metabolism to enhance immune response.
*B.*  *Based on Therapeutic Strategy/Level of Personalization:*
  1. Conventional Immunotherapy—broadly applicable agents such as cytokines and checkpoint inhibitors without patient-specific customization.
  2. Targeted/Precision Immunotherapy—therapeutics designed to precisely target tumor antigens or immune pathways, including antibody–drug conjugates, bispecific antibodies, and targeted cytokines.
  3. Personalized Immunotherapy—customized treatments based on individual tumor profiles, such as neoantigen vaccines, engineered TCR-T cells, and therapies guided by AI for patient-specific optimization.
  4. Combination/Next-Generation Immunotherapy—multimodal approaches combining immunotherapy with chemotherapy, radiotherapy, or advanced delivery systems (e.g., nanotechnology, AI-enabled platforms) to enhance efficacy.

### 3.1. Immune Checkpoint Inhibitors

The treatment of cancer with immune checkpoint therapy (ICT) has drastically transformed clinical outcomes (Table 2) for patients with cancer and conferred durable clinical benefits. Immune checkpoints play a key role in the regulation of the immune system’s network, preventing overactivation and autoimmunity [14]. This allows cancer cells to evade immune detection. Immune checkpoint inhibitors block the proteins that prevent the immune system from attacking the cancerous cells [15]. Therefore, targeting the immune checkpoint with inhibitors is a promising approach for cancer treatment.

#### 3.1.1. CTLA-4 Inhibitors

Cytotoxic T-lymphocyte antigen number 4 (CTLA-4) is a protein receptor found on T cells that prevents excessive immune activation and allows cancer cells to evade immune detection. Furthermore, other cells, including granulocytes, fibroblasts, and B cells, can also express it [16]. It was first discovered as an immune checkpoint in 1987. CTLA-4 competes with CD28 for interaction with B7 family proteins CD80/CD86 on antigen-presenting cells. CTLA-4 induces an inhibitory signal to T cells, while CD28 transmits a stimulatory signal. Immunosuppression results from CTLA-4’s binding to CD80/CD86 proteins, which blocks T-cells from recognizing and eliminating cancer cells [17]. CTLA-4 inhibitors are immunotherapy drugs that remove these brakes, allowing CD28 to activate T cells and enhance the immune system’s ability to fight cancer. Melanoma, liver cancer, lung cancer, and colorectal cancer are all treated with CTLA-4 inhibitors. Recent advancements with the development of nanobody-based strategies to inhibit CTLA-4 checkpoint could be a potential development in cancer immunotherapy [18].

#### 3.1.2. PD-1/PD-L1 Inhibitors

The Programmed Death-1 (PD-1) is a checkpoint that limits the immune system from targeting other cells in the body. It is present on the surface of T cells, expressed by B cells, monocytes and dendritic cells as well [19]. PD-1 on T cells binds to programmed cell death receptor ligand 1 (PD-L1) on cancer cells and inhibits T cell activation, hence leading to apoptosis of T cells, effectively stopping immunological action [20]. PD-1/PD-L1 inhibitors prevent the interaction between PD-1 and PD-L1, which removes the brakes and unleashes the immune system against the cancer cells. The FDA has approved six immune checkpoint inhibitors for this pathway, including pembrolizumab, nivolumab and cemiplimab for PD-1 and atezolizumab, avelumab and durvalumab for PD-L1 [21]. PD-1/PD-L1 inhibitors are being used with other inhibitors or therapies, offering a revolutionary advantage. PD-1/PD-L1 inhibitors, along with CTLA-4 inhibitors, have shown effective results for the treatment of melanoma and lung cancer [22].

#### 3.1.3. Emerging Checkpoint Targets (LAG-3, TIM-3, TIGIT)

**Figure 3:**
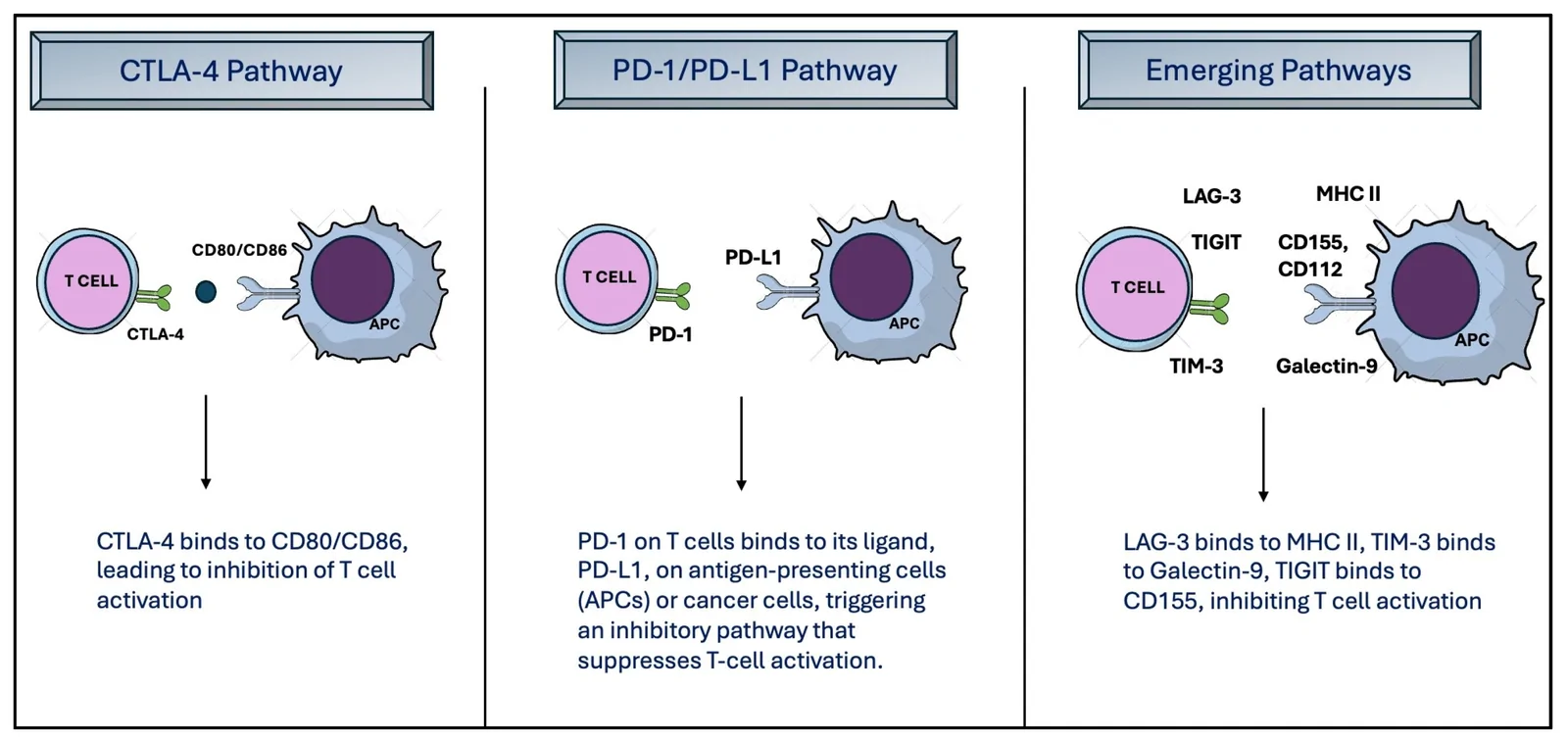
Pathways of Immune Checkpoint Inhibitors. The figure depicts key immune checkpoint pathways that inhibit T-cell function: CTLA-4 blocks early T-cell activation via CD80/CD86, PD-1/PD-L1 suppresses effector responses in peripheral tissues, and emerging checkpoints (LAG-3, TIM-3, TIGIT) further promote T-cell exhaustion and tumor immune evasion.

Though the anti-PD-1 and anti-CTLA-4 blockade showed a promising approach for cancer treatment, it is not for all patients with cancer, so there is a need for improvement. LAG-3, TIM-3, and TIGIT are emerging immune checkpoint targets, representing the next frontier in cancer immunotherapy [23]. Activated T and B cells, NK cells, and dendritic cells all express lymphocyte activation gene-3 (LAG-3), which is structurally homolog to the CD4 molecule [24]. LAG3 transmits inhibitory signals to T cells upon binding to MHC class II and other ligands, rendering T cells dysfunctional and maintaining immunological homeostasis [25]. Anti-LAG-3 and anti-PD-1 combination immunotherapy may be a potential approach in fighting PD-1 resistance [26]. T cell immunoglobulin and mucin-domain containing 3 (TIM3) enhances immune tolerance and its dysregulation leads to the development of autoimmune diseases [27]. Galectin-9, carcinoembryonic antigen-related cell adhesion molecule 1 (CEACAM1), phosphatidyl serine (PtdSer), and high-mobility group protein B1 (HMGB1) are its known ligands. TIM-3 in combination with other checkpoint inhibitors, including PD-1, is being developed and is currently under examination [28]. Moreover, a transmembrane protein composed of a type 1 transmembrane domain (TIGIT) is another emerging key inhibitor of anti-tumor responses that can impede several stages of the cancer immunity cycle. TIGIT competes with the receptor CD226 or CD96 for binding to shared ligands CD155 and CD112. Where CD226 offers a positive co-stimulatory signal, while CD96 and TIGIT exhibit inhibitory signals [23]. This binding of TIGIT to its ligand results in the inhibition of the activity of T cells and NK cells, causing an overall immune suppressive response. TIGIT inhibitors in combination therapy with anti-PD-1/PD-L1 for various cancer treatments, including NSCLC, are currently being evaluated in clinical trials [29]. Thus, these three targets hold promise for improved outcomes of immunotherapy (Figure 3).

### 3.2. Adoptive Cell Therapies

**Figure 4:**
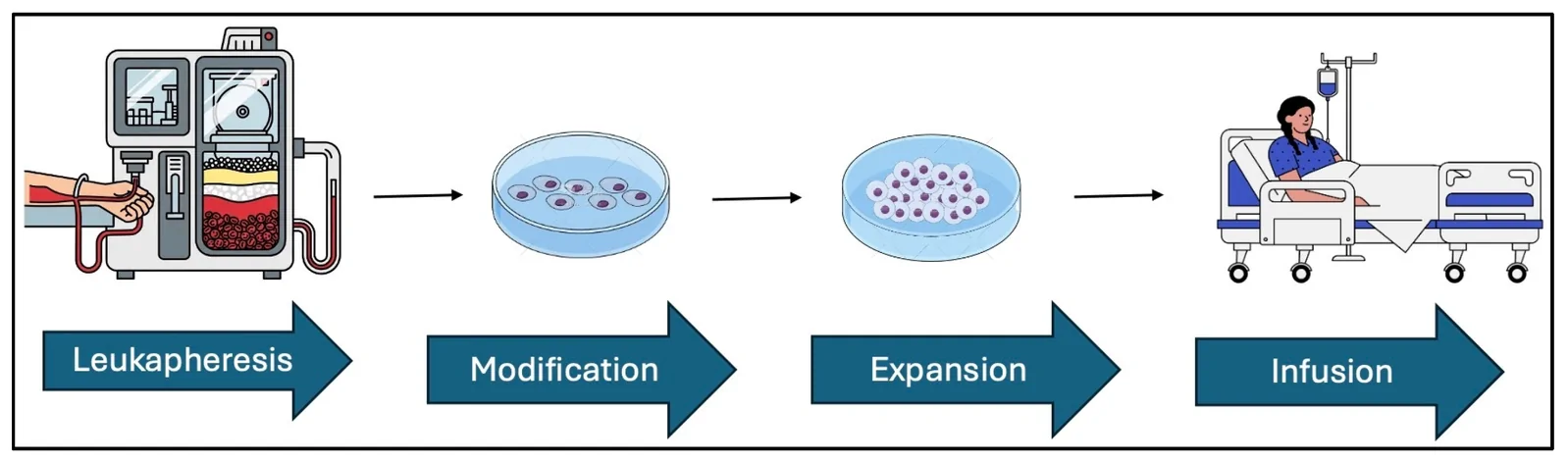
Workflow of adoptive cell therapy.

Adoptive cell immunotherapy (ACT) is a vibrant field of immunotherapy where the immune cells are extracted from a patient, multiplied or genetically modified outside the body and then reinfused back into the patient to fight cancer cells (Figure 4) The main types of adoptive cell therapies are as follows:

#### 3.2.1. CAR T-Cell Therapy

Chimeric antigen receptor (CAR) T-cell immunotherapy is one of the most promising types of adoptive cell therapies. It involves genetic modification of the patient’s T cells to express a chimeric antigen receptor to fight cancer cells [30]. CARs have the advantageous ability to recognize antigens or proteins on cancer cells, even without their presentation via MHC [31]. It allows bypassing the immune suppression caused by the downregulation of MHC-associated antigens on cancerous cells. In 2017, the U.S. FDA approved the first CAR-T therapy for the treatment of B-cell malignancies. However, despite the potential effect in the treatment of B-cell lymphomas and leukemia, CAR-T therapy for solid tumors is still under evaluation. To date, after successful clinical trials, the FDA has approved six CAR-T cell products (Table 3) for the treatment of hematological malignancies [32].

#### 3.2.2. Tumor-Infiltrating Lymphocyte Therapy

Tumor-infiltrating lymphocytes (TIL) are the lymphocytes that are found inside the tumor. TIL therapy involves the culture of lymphocytes collected from a surgically resected tumor, expanded in number in vitro, and then reinfused back into the patient [33]. The FDA approved the first TIL therapy, i.e., lifileucel, in 2024 for the treatment of solid tumors, skin cancer, and melanoma [34]. Initially, TIL therapy was considered a niche treatment that would have efficacy only against melanoma, while now, it is becoming a promising approach for a growing number of tumor types [33].

#### 3.2.3. TCR-Engineered T Cell Therapy

TCR-engineered T cells (TCR-T cells) immunotherapy uses engineered T lymphocytes that express T cell receptors designed to recognize and destroy specific cancer antigens. It differs from CAR-T cell therapy, as TCR-T cells can recognize antigens expressed both on cell surfaces as well as in intracellular compartments [35]. It is gaining attention and rapidly growing because it may be an effective strategy to treat solid tumors [36]. In August 2024, the USA FDA approved the first TCR-T therapy called TECELRA or afami-cel for the treatment of metastatic synovial sarcoma [37]. Moreover, the precise identification and selection of TCRs that are both effective and safe are crucial for the success of TCR-T cell therapies for cancer treatment.

#### 3.2.4. NK Cell-Based Therapy

Natural killer cells are an essential component of innate immune response, which can identify and destroy cancer cells without prior exposure, making them a potential candidate for immunotherapeutic applications. Current advances have impactfully increased the therapeutic capacity of NK cells by enhancing their recognition and cytotoxic abilities [38]. In NK-based therapy, autologous (patient’s own) or allogenic (healthy donor) NK cells are isolated, expanded, and infused back into the patient. Both of these NK cells can be further genetically modified, known as CAR-NK cells, expressing CARs targeting specific tumor antigens to enhance their cancer-killing abilities. NK cell-based therapy alone or in combination with other therapies is currently under clinical trials [39].

### 3.3. Cancer Vaccines

**Figure 5:**
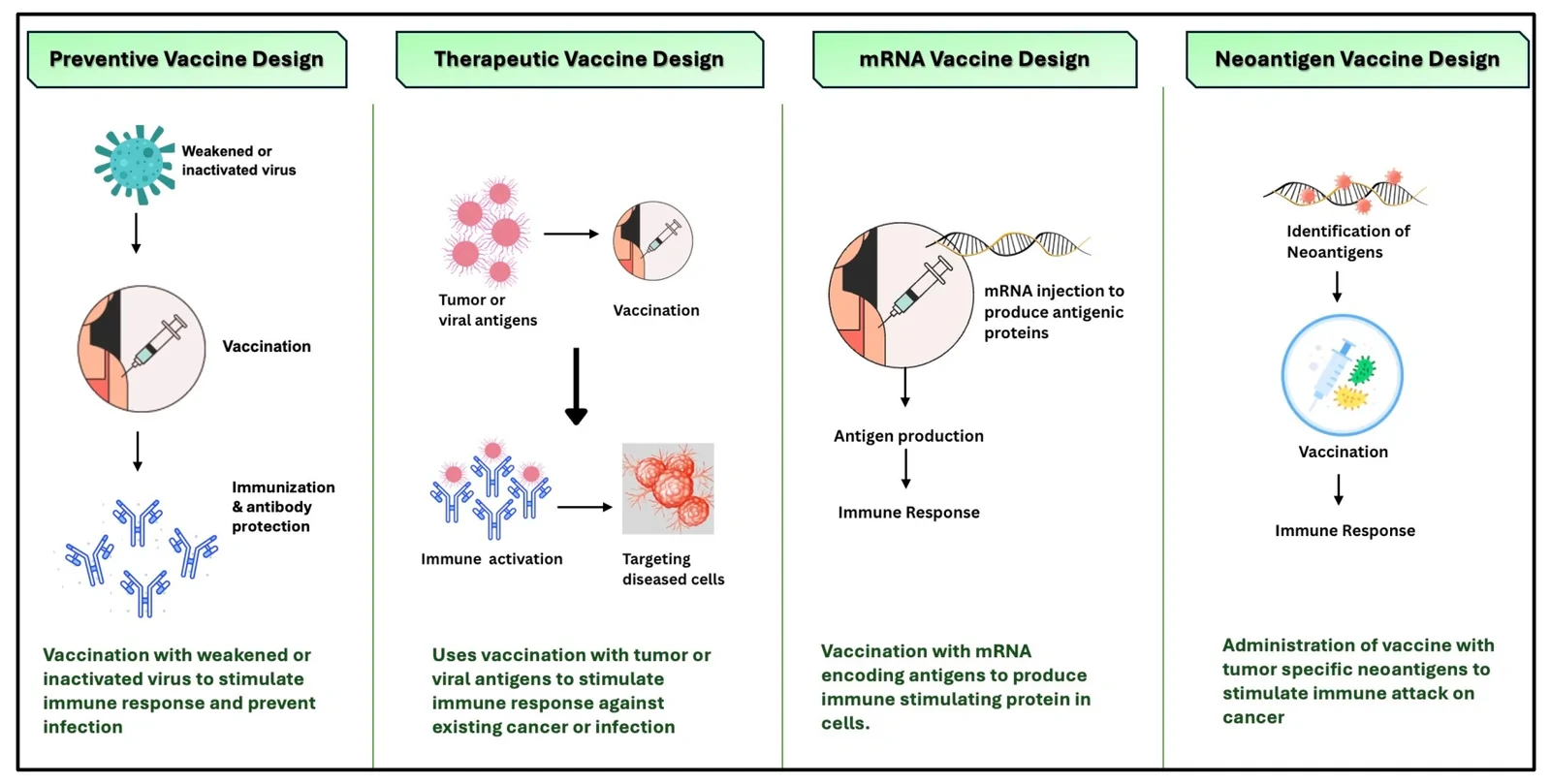
Types of cancer vaccines and their mechanisms of action.

Cancer vaccines are a form of immunotherapy that stimulate the immune system to identify and eliminate cancer cells for the prevention or treatment of cancer (Figure 5). The following are the different types of cancer vaccines:

#### 3.3.1. Preventive Cancer Vaccines

Preventive cancer vaccines aim to prevent cancer before it develops by stimulating the immune system to detect and destroy the cancer-causing agents and pre-cancerous cells. Where several cancers are developed because of viral infections and preventive cancer vaccines facilitate the body’s immune system to fight against these cancer-causing viruses. These vaccines reduce the risk of infections and the consequent development of cancer in the body. To date, several vaccines have been created, including Cervarix, Gardasil, and Hepatitis B, to protect against malignancies as well as infections caused by HBV and HPV [40]. This field is rapidly growing to develop vaccines beyond viral protection for broader cancer prevention [41].

#### 3.3.2. Therapeutic Cancer Vaccines

A therapeutic cancer vaccine aims to boost the immune system to recognize and destroy cancer cells. Unlike preventive vaccines that aim to stop cancer before it has developed in the body, therapeutic vaccines are designed to control or eliminate the existing cancer. The two main types of this vaccine are patient-specific and patient non-specific [42]. There are different approaches to therapeutic cancer vaccines such as cell-based vaccines, peptide-based vaccines, virus-based vaccines and gene-based vaccines (Table 4). Some FDA-approved therapeutic cancer vaccines are Sipuleucel-T for metastatic prostate cancer, Bacillus Calmette–Guérin (BCG) and Nadofaragene firadonevec (Adstiladrin) to treat early-stage bladder cancer and T-VEC (Imlygic) for melanoma [43].

#### 3.3.3. mRNA Vaccine Platforms

Messenger RNA (mRNA) vaccines have arisen as a revolutionary platform in modern vaccinology. This approach uses mRNA to instruct cells to produce proteins, acting as antigens to elicit an immune response. The key components of an mRNA vaccine are: the mRNA strand, which encodes the antigen, 5′ cap structure, 5′ and 3′ UTRs, and Poly(A) tail. In this process, mRNA is encapsulated in a delivery system, often lipid nanoparticles, for protecting and facilitating its delivery into the cell. These platforms deliver rapid development capabilities, high efficacy, and versatility across multiple disease targets. During the COVID-19 pandemic, mRNA vaccines gained global prominence, but they continue to face challenges in stability, delivery, and immune response optimization [44]. Ongoing research continues to address the limitations of this technology.

#### 3.3.4. Personalized Neoantigen Vaccines

Personalized neoantigen vaccines are a form of cancer immunotherapy that harnesses the power of the immune system to destroy the cancer cells by targeting unique mutations found in a patient’s tumor. These vaccines target specific neoantigens, which are novel protein fragments that arise from mutation in cancer cells and are absent in normal tissues. The previous study has reported that recurrence did not occur after two years in over 60% of patients with melanoma treated with personalized tumor neoantigens [45]. Additionally, the efficacy of these vaccines strongly relies on the good delivery methods, like encapsulation of neoantigens in lipid nanoparticles and viral vectors [46].

### 3.4. Monoclonal Antibodies

**Figure 6:**
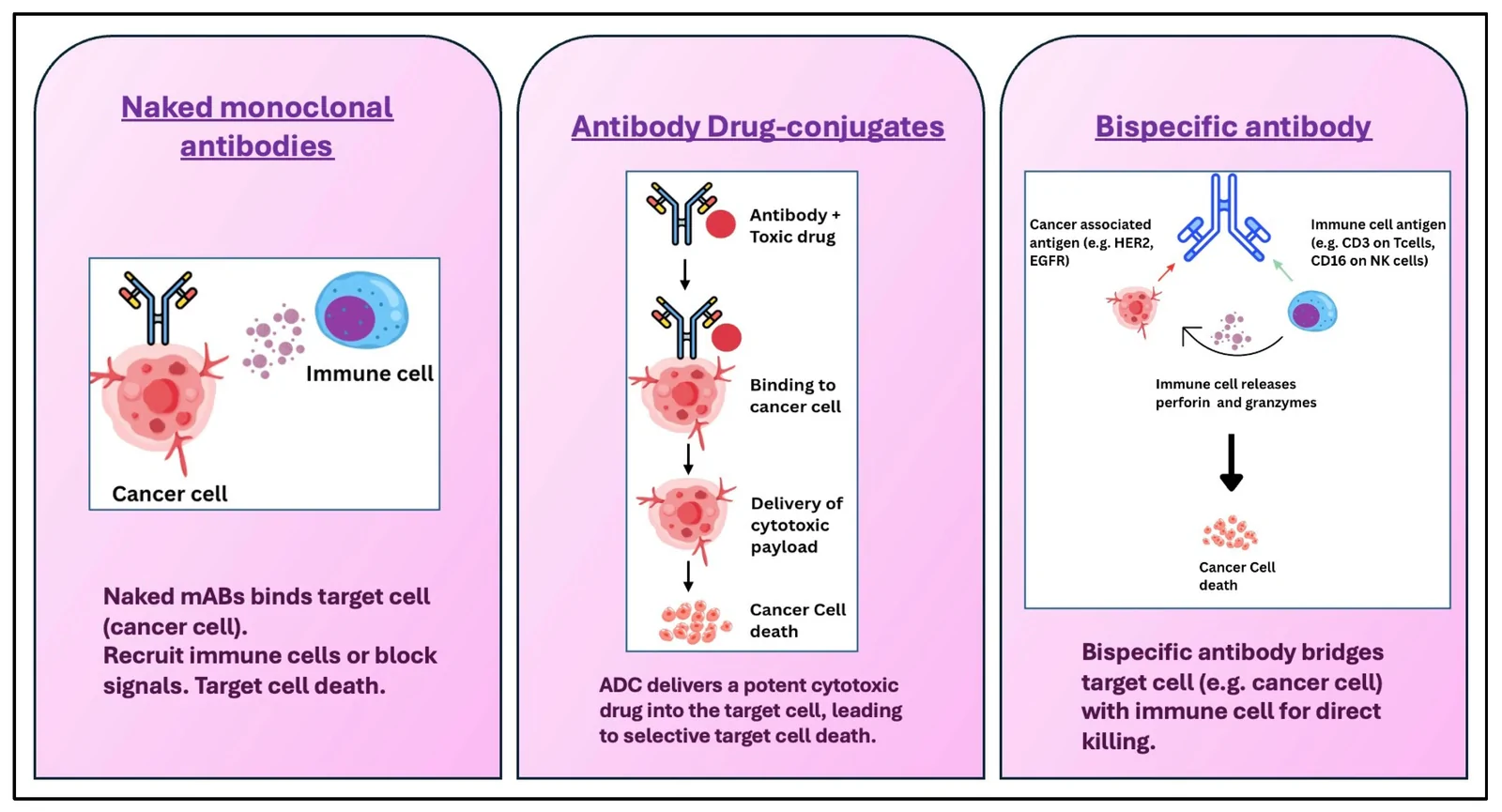
Mechanistic comparison of antibody-based cancer therapeutics.

Monoclonal antibodies (mAbs) are lab-produced antibodies designed to target a specific antigen. They are generated by cloning a single type of immune cell that specifically targets one epitope. For cancer treatment, ninety-one monoclonal antibodies are approved in clinics, either alone or in combination with other antibodies or chemotherapeutic agents [47]. The therapeutic efficacy of these antibodies, due to their multifaceted properties, offered a major impact on cancer treatment strategies (Figure 6). The common types of monoclonal antibodies used for cancer treatment:

#### 3.4.1. Naked Monoclonal Antibodies

Naked mAbs are unconjugated antibodies, not linked with any drug or radioactive substance. They act independently through direct binding to specific targets on cancer cells. These antibodies can stimulate immune responses or inhibitory signal pathways. They are widely and most commonly used mAbs in cancer immunotherapy [48]. These antibodies have several distinct mechanisms in cancer therapy, including immune system recruitment, immune checkpoint modulation and signal transduction blockade. They have fewer serious side effects than chemotherapeutic drugs and are highly specific. In 1997, the FDA approved rituximab for leukemia as the first naked mAbs for cancer immunotherapy. Since then, many naked mAbs have received FDA approval [49] (Table 5).

#### 3.4.2. Antibody-Drug Conjugates

Antibody–drug conjugates (ADCs) are a type of cancer immunotherapy that combines a tumor-specific monoclonal antibody with a cytotoxic drug, delivered via a chemical linker. They deliver wider therapeutic openings and improved pharmacokinetic/pharmacodynamic properties by efficiently targeting the cancer cells while limiting harm to healthy tissue [50]. When ADC binds to its target antigen on cancer cells, it enters the cells and the payload is released, which disrupts the crucial cellular processes and ultimately leads to tumor cell death [51]. Combining ADC with other cancer immunotherapies has a potential therapeutic effect in solid tumors and in overcoming resistance mechanisms. A recent study reveals that the combination of ADC and ICI therapeutics might be a promising treatment option [52]. Also, recent evidence suggests that certain maytansine-bearing antibody–drug conjugates can induce hallmarks of immunogenic cell death (ICD) in antigen-positive tumor cells. Such ICD-mediated immune activation may contribute to improved responses to immune checkpoint inhibitors, including anti-PD-L1 therapies. Hence, induction of immunogenic cell death (ICD) has emerged as a promising strategy in cancer immunotherapy for enhancing anti-tumor immune responses and modulating the tumor immune microenvironment [53]. Trastuzumab emtansine (T-DM1), trastuzumab deruxtecan (T-DXd), Sacituzumab govitecan (SG), and datopotamab deruxtecan (Dato-DXd) have been recently approved by the FDA for breast cancer treatment.

#### 3.4.3. Bispecific and Multispecific Antibodies

Bispecific and multispecific antibodies are engineered molecules, allowing them to simultaneously target two or more antigens or epitopes [54]. Bispecific antibodies (BsAbs) have two different binding domains that can simultaneously bind to two antigens or two epitopes of the same antigen. Multispecific antibodies (msAb) are antibodies that can bind to three or more different targets or epitopes. The property of these antibodies to bind simultaneously to different epitopes enhanced their therapeutic efficacy in cancer treatment and infectious diseases as well. They can direct immune effector cells (like T-cells) to tumor cells, leading the immune system to attack diseased cells. The development and manufacturing of these antibodies comprises multiple challenges, including complex assembly, stability, immunogenicity, and the requirement for sophisticated analytical and production methods [55].

### 3.5. Tumor Microenvironment Modulation

**Figure 7:**
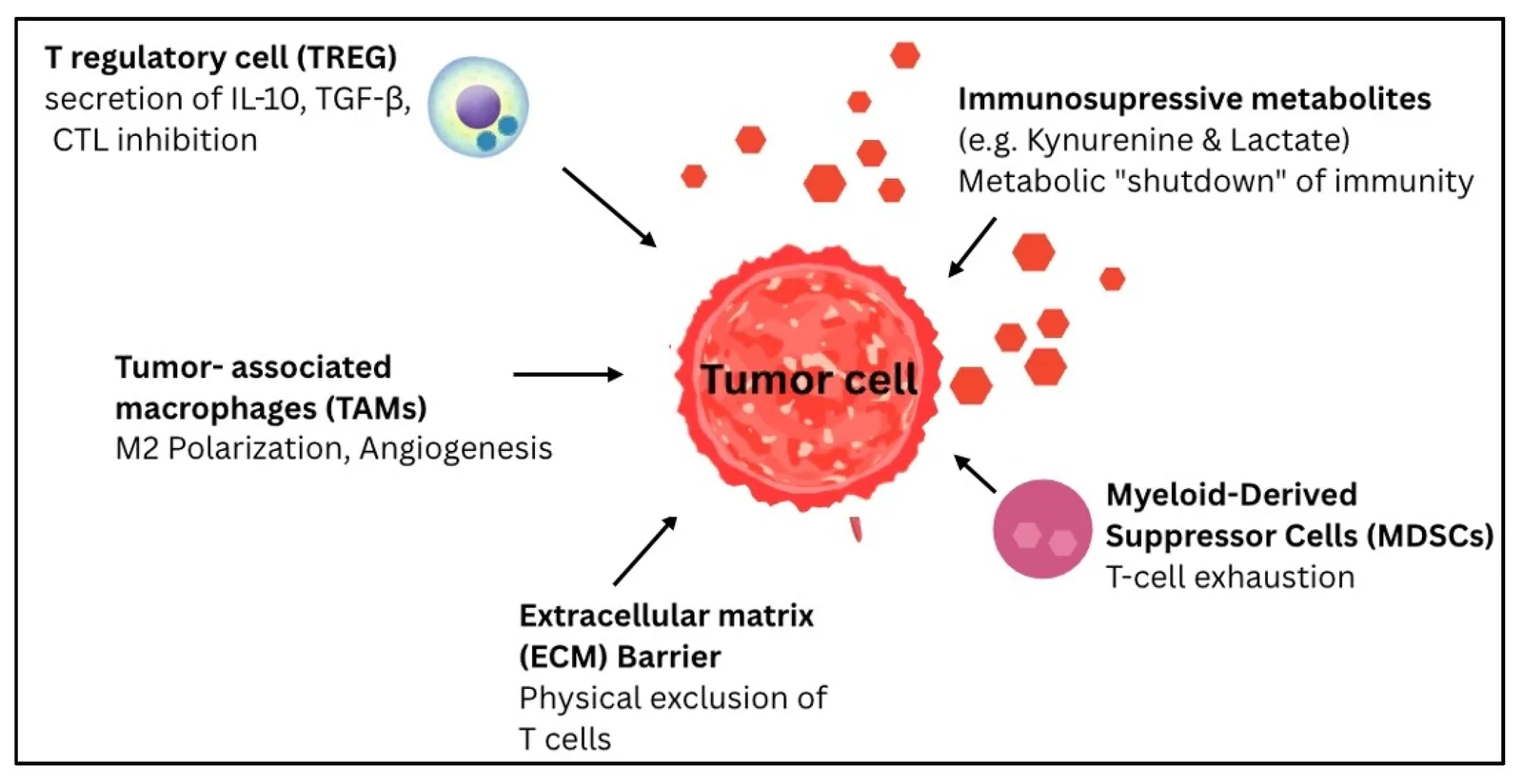
Immunosuppressive TME components and therapeutic interventions. The figure depicts Tregs, TAMs, MDSCs, inhibitory cytokines/metabolites, and ECM barriers, which collectively suppress anti-tumor immune responses and limit T-cell activity.

The tumor microenvironment (TME) represents a complex ecosystem that surrounds tumor cells that significantly has a major impact on both cancer progression and therapeutic responses. TME modulation is the approach to overcome immunosuppression and enhance the therapeutic outcome of immunotherapy (Figure 7). There are different strategies for the alteration of the tumor microenvironment surrounding the tumor cells.

#### 3.5.1. Targeting Immunosuppressive Cells

Several immunosuppressive cells, including myeloid-derived suppressive cells (MDSCs), tumor-associated macrophages (TAMs), tumor-associated neutrophils, regulatory T cells (Tregs), and tumor-associated dendritic cells, hinder anti-tumor response [56]. These immunosuppressive cells hinder the anti-tumor immunity through different mechanisms like metabolic reprogramming, secretion of cytokines and by inducing inhibitory signals [56]. Targeting these immunosuppressive cells through the use of peptides and peptide-based nanomedicine, blocking the immune checkpoint, and reprogramming these cells are promising therapeutic approaches (Table 6). However, the production and activation of immunosuppressive cells within the TME comprises complex mechanisms, which makes it challenging for a single therapeutic strategy to produce strong anti-tumor effects. Thus, immunosuppressive cell-targeted therapy in combination with other anti-tumor therapies will be a promising strategy.

#### 3.5.2. Modulating Stromal Components

Modulating stromal components is one of the major evolving areas of research in cancer immunotherapy. It involves targeting the extracellular matrix (ECM) and stromal cells, aiming to overcome the major barriers presented by the TME, to improve the efficacy of cancer treatment. Stromal cells are crucial elements of TME, including cancer-associated fibroblasts (CAFs), endothelial cells, immune cells, mesenchymal stem cells and pericytes. These cells play an important role in the occurrence and growth of tumors and drug resistance as well. Various therapeutic approaches employ their effects on stromal components through different mechanisms. ECM remodeling by enzymatic degradation (collagenase, hyaluronidase) or physical disruption can decrease its density and stiffness, improving immune cell response [57]. Inhibiting ECM production by blocking TGFβ signaling and blocking ECM stiffness using the enzyme lysyl oxidase (LOX) improves tumor perfusion and facilitates T cell infiltration. Monoclonal antibodies like STRO-501, targeting BIGH3, are being developed to reduce stroma complexity and prepare tumors for surgical resection. Accordingly, modulation of stromal components improves tumor infiltration of cytotoxic T cells, enhances the response rates to immune checkpoint inhibitors, and allows effective penetration of therapeutic agents.

#### 3.5.3. Metabolic Reprogramming Strategies

Metabolic reprogramming strategies in cancer immunotherapy involve manipulating the metabolic environment of both immune and tumor cells within the TME to enhance anti-tumor immunity. According to a prior study, several metabolic regulators interact with immune cells to build a metabolic network inside the TME, which guides the tumor immune response [58]. Cancer cells manipulate the metabolism to fuel their rapid growth and survival, where metabolic dysregulation creates an immunosuppressive TME that can suppress anti-tumor immunity. The main approaches include targeting cancer cell metabolic pathways, reprogramming the tumor microenvironment (TME), and developing metabolic interventions, such as enzyme inhibitors and nanomedicines and combination therapies to augment immune cell activity and combat treatment resistance. Advanced genetic engineering tools like CRISPR/Cas9 can also be used for metabolic reprogramming to enhance the immunotherapy effectiveness [59].

#### 3.5.4. Interaction of Coagulation and Immunological Factors in the Tumor Microenvironment

Hypercoagulability is widely acknowledged as a hallmark of cancer progression and immune modulation. Shortened activated partial thromboplastin time (aPTT), a marker of increased thrombin generation and elevated coagulation factors, has been shown to reflect a prothrombotic state in physiological conditions such as pregnancy [60]. Similarly, malignancies induce a hypercoagulable microenvironment through tumor-derived tissue factor, inflammatory cytokines, and endothelial activation. Coagulation proteases such as thrombin not only promote thrombosis but also modulate immunological responses within the tumor microenvironment (TME), influencing macrophage polarization, T-cell suppression, and tumor progression. Thus, the coagulation system represents an underexplored immunomodulatory axis in cancer immunotherapy.

### 3.6. Cytokine Therapies

Cytokines are small proteins secreted by immune cells that act as chemical messengers to regulate immune response, inflammation, and hematopoiesis. Cytokine therapies to develop cancer treatments, leveraging the immune system’s natural signaling molecules to enhance anti-tumor immunity, have gained interest over the years. Studies have shown the therapeutic anti-tumor activity of cytokines in murine models and in the clinical application of some human cancers [61]. While only a few cytokines have received FDA approval as monotherapies (Table 7), research is evolving rapidly, mainly in combination therapies and engineered cytokines [62]. Several exogenous cytokines or cytokine-modulating agents are used to enhance anti-tumor immune responses.

#### 3.6.1. Interleukins

**Figure 8:**
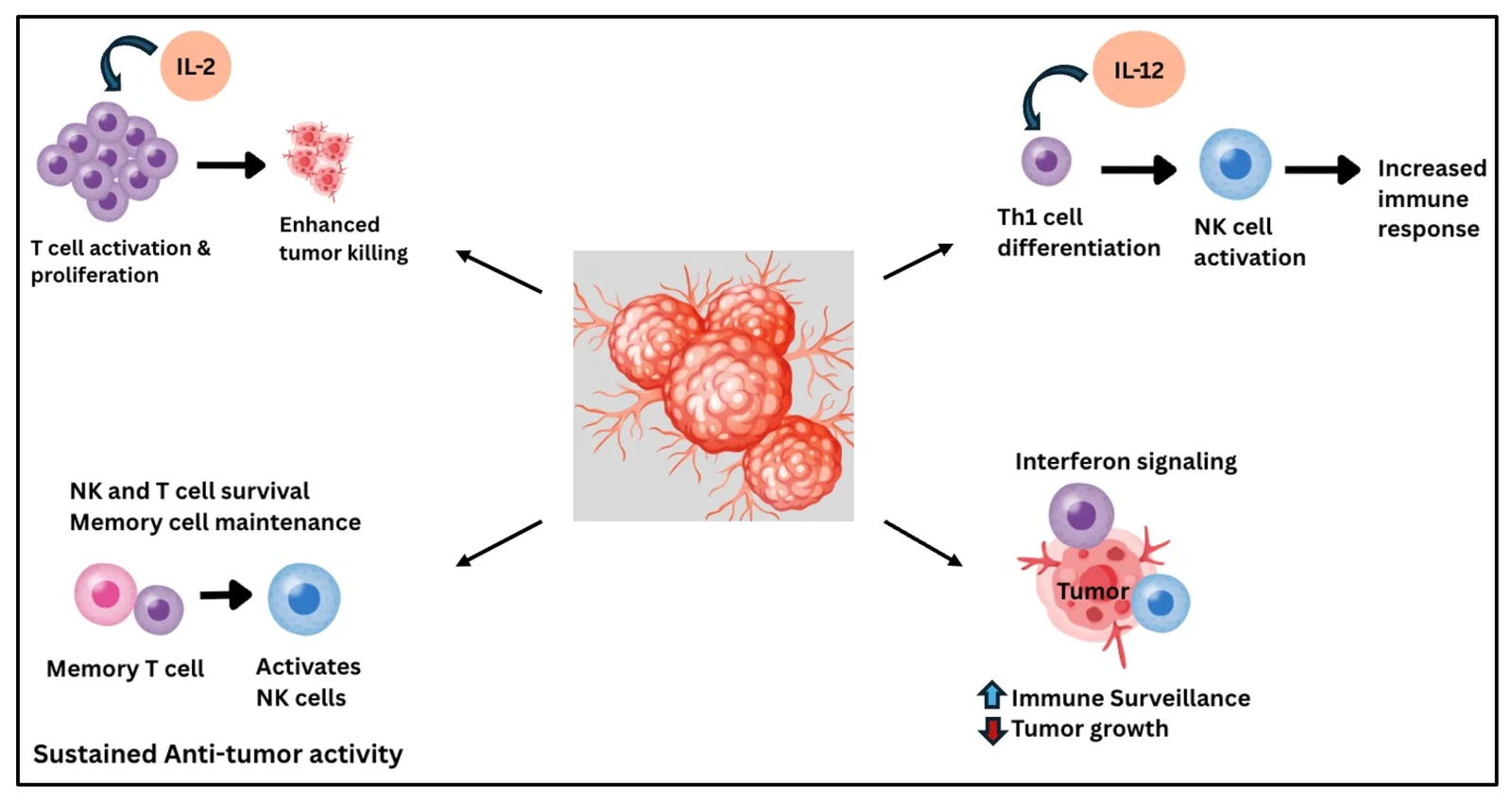
Roles of IL-2, IL-12, IL-15, IFNs in anti-tumor response.

Interleukins (ILs) are cytokines that play critical roles in cancer immunotherapy and modulate the immune cell activity by either stimulating or suppressing tumor growth (Figure 8). Interleukins like IL-2, IL-12, and IL-15 activate anti-cancer immune cells such as T cells and NK cells to enhance anti-tumor activity, whereas other interleukins, including IL-6 and IL-8, suppress anti-tumor immunity and can promote tumor development, making them suitable therapeutic targets. IL-2 was the first interleukin approved by the U.S. FDA for cancer treatment and remains a cornerstone of cancer immunotherapy. It has been used for the treatment of metastatic melanoma and renal cell carcinoma [63]. IL-12 can activate both innate and adaptive immunity by promoting Th1 responses, IFN-γ production and enhancing the cytotoxic activity of T cells and NK cells. Preclinical studies demonstrated its anti-tumor activity; however, its clinical trials in the late 1990s/early 2000s showed disappointing outcomes due to its severe toxicities [64]. This led the researchers to focus on approaches to deliver IL-12 directly to the tumor site or in a controlled, localized manner. IL-15 also plays an important role in cancer immunotherapy, by activation of natural killer (NK) cells and CD8+ T cells, and IL-15-based immunotherapies are currently undergoing clinical trials [65]. Moreover, researchers are developing strategies, like engineered variants and combination therapies, to augment the efficacy of IL-based immunotherapies and minimize side effects.

#### 3.6.2. Interferons

Interferons (IFNs) are signaling proteins that act as key cytokines in cancer immunotherapy, modulating both innate and adaptive immune regulation. IFNs exhibit both anti-tumor and pro-tumor effects; chronic low-level interferon signaling promotes tumor progression and resistance to therapy, while acute, robust interferon leads to immune activation and elimination of cancer cells. There are three main types of interferons: type I (IFN-α, IFN-β), II and III. Type I (IFN-α, IFN-β) interferons, produced by many cell types, directly prevent tumor cell growth, trigger apoptosis, and make cancer cells more sensitive to treatment. IFN-α was the first FDA-approved human immunotherapeutic agent for treating hairy cell leukemia [66]. Type II interferon (IFN-γ), predominantly produced by T cells, promotes immune identification and tumor cell removal, but it can also trigger immunological checkpoint molecules like PD-L1, which results in immune evasion. Type III (IFN-λ) could be a better choice for disease targeting and minimizing side effects, due to its restricted cell response pattern. Even though it is considered to have a potential role in cancer treatment, limited clinical trials have been conducted for its use in immunotherapy [67]. Here, IFN-based immunotherapies have demonstrated efficacy in different cancers, but toxicity, pleiotropic effects, and immune escape potential limit their practical application.

#### 3.6.3. Engineered Cytokines and Cytokine Delivery Systems

Engineered cytokines and a cutting-edge cytokine delivery system have revolutionized the field of cancer immunonotherapy by maximizing the immune responses while reducing toxicity and off-target consequences. Numerous modifications have been developed for enhancing tumor targeting and prolonging the half-life of cytokines, including polyethylene conjugation, fusion to antibodies and modifications in the cytokine/cell receptor-binding affinity [68]. Furthermore, for enhanced bioavailability and reduced systemic toxicity, cytokines can be delivered directly into the tumor microenvironment using localized delivery platforms such as hydrogel depots or nanoparticles [65,69,70]. A novel engineered IL-15 superagonist, N-803 (Anktiva), approved by the FDA for the treatment of non-invasive bladder cancer, indicates the potent clinical application of engineered cytokines for cancer immunotherapy [71]. Another immunocytokine, NHS-IL12, has been designed for delivery of IL-12 to the tumor microenvironment (TME) [72].

### 3.7. Oncolytic Virus Therapy

Oncolytic virus therapy represents an emerging approach to cancer treatment that uses the naturally occurring or genetically engineered viruses to selectively infect and eliminate cancer cells while boosting anti-tumor immunity.

#### 3.7.1. Mechanism of Action

**Figure 9:**
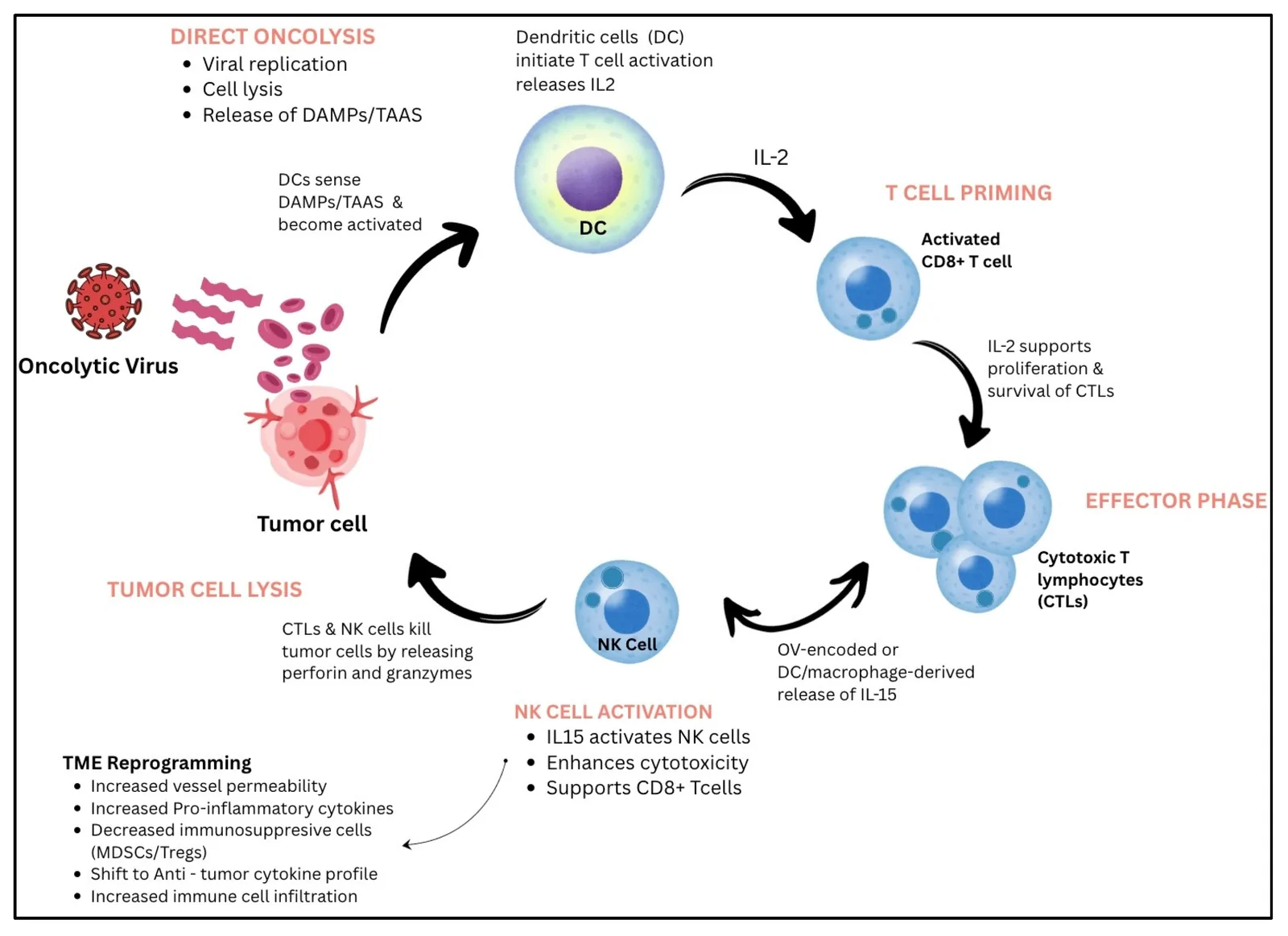
Mechanism of OV therapy.

Oncolytic virus therapy works through different mechanisms, including direct oncolysis, provoking a systemic anti-tumor immune response, tumor microenvironment reprogramming, genetic alterations and in combination with other therapies. Direct oncolysis represents the mechanism where the virus selectively infects and replicates within the cancer cells, leading to lysis of cancer cells through different mechanisms (Figure 9), without harming the normal cells. In addition to tumor destruction, cancer cell lysis releases tumor-associated antigens (TAAs), cellular damage-associated molecular patterns (DAMPs) and pathogen-associated molecular patterns (PAMPs), creating an inflammatory tumor microenvironment [73]. This process is described as immunogenic cell death, where a cold immunologically silent tumor turns into a hot one that is more susceptible to immune clearance. Oncolytic viruses (OVs) can reprogram the TME activation of innate and adaptive immune cells, remodeling the tumor metabolism, increasing production of chemokines and cytokines that improve immune cell infiltration, and enhancing antigen presentation [74]. Moreover, using oncolytic virus therapy in combination with other treatments like immune checkpoint inhibitors, CAR-T cell therapy and chemotherapy can enhance the therapeutic efficacy (Table 8).

#### 3.7.2. Clinical Development Status

Oncolytic virus therapy has shown promising anti-tumor effects and is in the growing stage in clinical development for cancer immunotherapy. The first oncolytic virus approved by the FDA was Talimogene laherparepvec (T-Vec), a genetically modified herpes simplex virus (HSV-1), expressing GM-CSF, approved for advanced melanoma [75]. It showed significant effectiveness both as monotherapy and in combination with other immunotherapies as well. Teserpaturev (Delytact) received conditional approval in Japan for the treatment of patients with malignant glioma [76]. In 2005, the Chinese FDA approved Oncorine for nasopharyngeal carcinoma in combination with chemotherapy [77]. In a study, a phase 1/2 trial demonstrated that the intratumoral delivery of oncolytic virus DNX-2401 in combination with intravenous anti-PD-1 antibody pembrolizumab is safe and provides survival benefits in recurrent glioblastoma [78]. Furthermore, oncolytic viruses are being tested with various other immunotherapies to enhance their therapeutic efficacy and promote clinical application. Many oncolytic viruses are under clinical trial for the treatment of head and neck cancer [77].

#### 3.7.3. Engineering Strategies to Enhance Efficacy

Natural oncolytic viruses can destroy the tumor cells, but they have limited clinical activity. To enhance the clinical efficacy of oncolytic viruses in cancer immunotherapy, engineering strategies aim to improve the tumor selectivity, delivery and immunogenic stimulation. Genetic engineering of OVs is employed through approaches like introducing tumor-selective promoters and deleting virulence genes, armed with immune-stimulatory transgenes, and gene editing through CRISPR-Cas9 [79]. Aiming for enhanced therapeutic efficacy, OVs are modified to express the molecules that reprogram the immunosuppressive TME and increase antigen presentation [80]. Cellular carriers and nanotechnology-based delivery are being used for improved stability, bioavailability and reduced immune clearance [79]. A combination of oncolytic viruses with other immunotherapies like immune checkpoint blockade, antigen-specific T-cell receptors (TCRs), chimeric antigen receptors (CARs) and autologous tumor-infiltrating lymphocytes (TILs) has made significant progress in the enhancement of cancer treatment [81].

## 4. Clinical Applications

**Figure 10:**
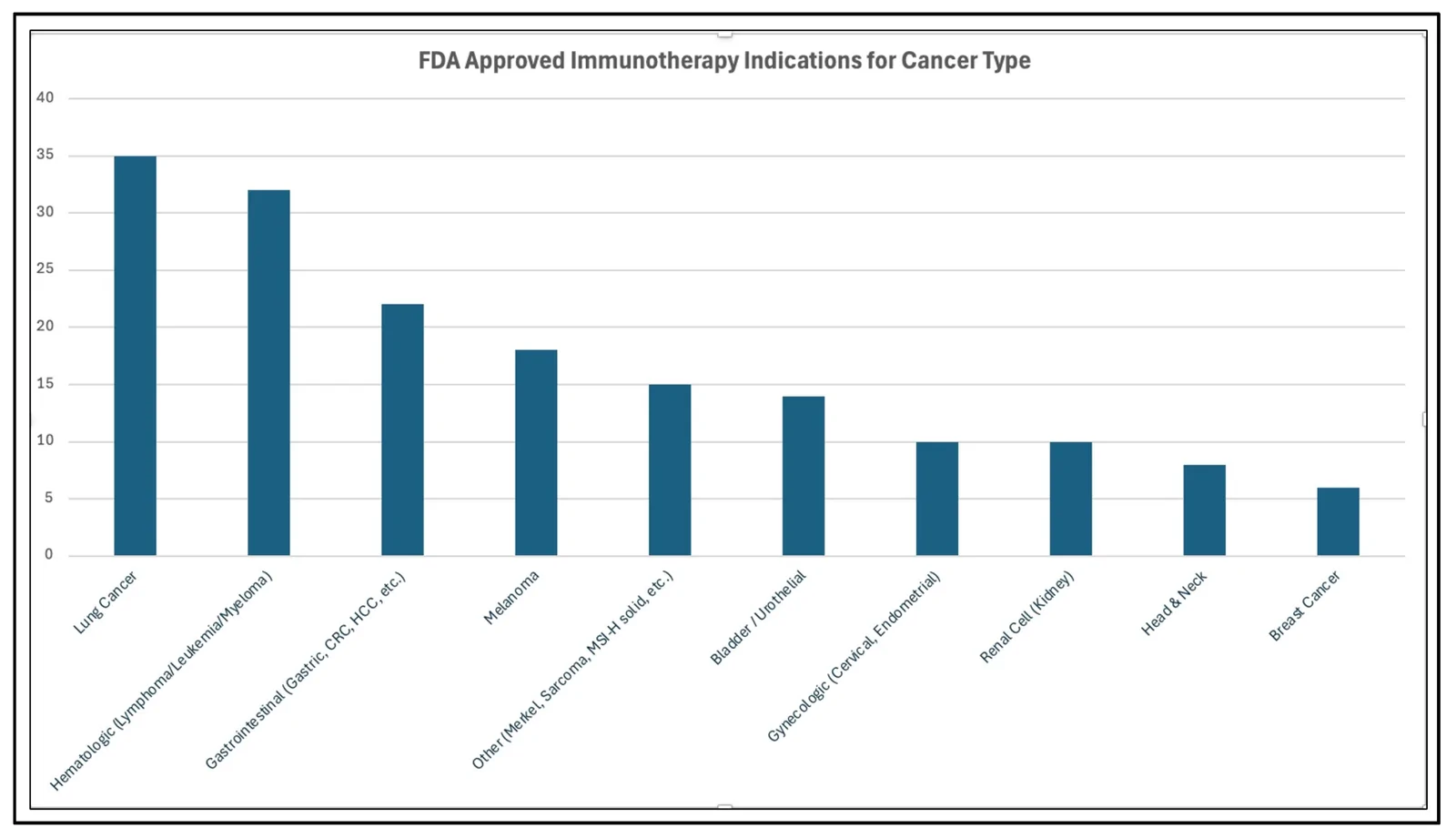
Distribution of FDA-approved cancer immunotherapies by cancer type. Bar chart showing the number of approved immunotherapeutic indications across major malignancies.

Immunotherapies for cancer have evolved over time and have moved from experimental conduct to clinical application across several cancers, offering hope for patients suffering from this deadly disease (Table 9). Clinical implementation of cancer immunotherapies comprises various therapies, including immune checkpoint inhibitors, adoptive cell therapies, monoclonal antibodies, cancer vaccines, tumor microenvironment modulation, cytokine therapies, and oncolytic virus therapies to harness the body’s own immune system to fight against multiple cancers [82]. However, these therapies have some limitations, and to overcome their challenges, combination therapies have revolutionized the use of immunotherapies in cancer treatment and have shown promising outcomes [83]. Successful immunotherapy relies on appropriate patient selection, where using biomarkers for patient selection that are appropriate to respond to immunotherapy will facilitate the clinical outcomes of the therapeutics. The predictive biomarkers PD-L1 expression, microsatellite instability (MSI), and tumor mutational burden (TMB) play a crucial role in the patient’s selection for ICI treatment [84]. Immunotherapies are being applied for the treatment of a wide range of cancers (Figure 10). The application of immunotherapy for high-risk melanoma has significantly increased the survival rate and reduced the risk of recurrence [85]. In addition, combination therapy with ICIs, ipilimumab and nivolumab has shown a synergistic effect on the immunity against the melanoma [85]. ICIs, PD-1/PD-L1 inhibitors, either as monotherapy or combined with chemotherapy, have demonstrated striking benefit in the survival of patients with non-small cell lung cancer [86]. Hematological malignancies have also benefited from different immunotherapies, such as CAR-T cell therapies and checkpoint inhibitors, which have led to better and long-term response rates [87]. Immunotherapy has also shown a promising therapeutic effect on renal cell carcinoma, with nivolumab in combination with ipilimumab becoming standard first-line treatments for many patients [88].

## 5. Benefits and Opportunities of Immunotherapy in Cancer

**Figure 11:**
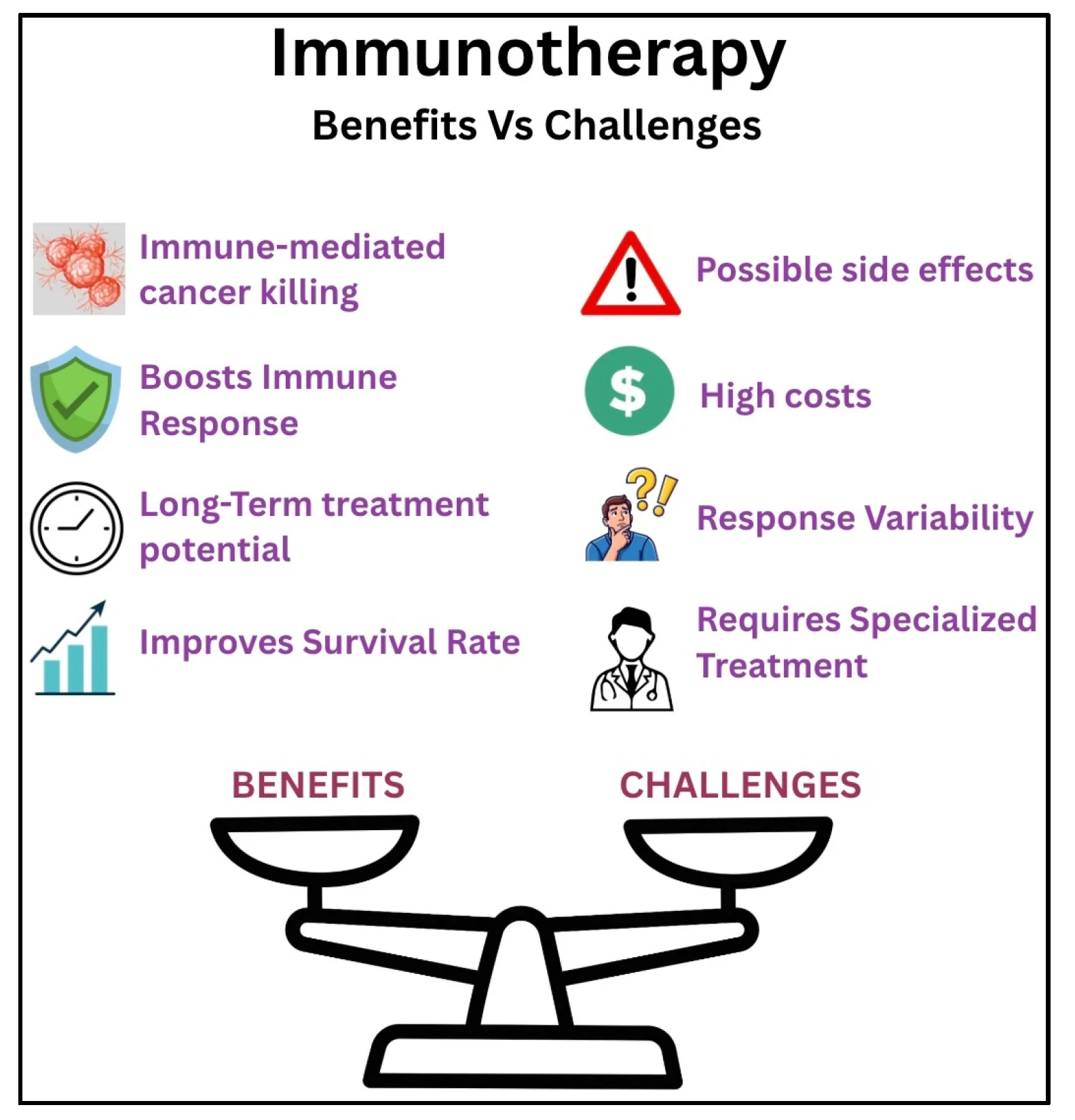
Benefits versus challenges associated with cancer immunotherapy.

Immunotherapy signifies a paradigm shift in therapeutic approaches by utilizing the body’s own immune system to recognize and eliminate cancer cells (Figure 11). Several clinical studies have shown that immunotherapy is advantageous over traditional therapies for cancer treatment, by offering prolonged survival [95]. Cancer cells often develop mechanisms to escape from the immune system, termed immune evasion. Immunotherapies, such as ICIs, including PD-1/PD-L1 and CTLA-4 inhibitors, help to overcome this evasion and allow the immune system to recognize and kill cancer cells. Immunotherapy can target and eliminate specifically the cancer cells while causing minimal damage to surrounding healthy cells, offering precise treatment [96]. Unlike traditional therapies, immunotherapy delivers long-lasting survival and remission, where the immune response continues even after the end of the treatment and reduces the risk of recurrence [97]. Another benefit of immunotherapy is that it is associated with fewer side effects compared to chemotherapy and radiation, offering improved patients’ quality of life [96]. One of the main benefits of immunotherapy is that it has shown success in treating a broad range of cancers, including melanoma, lung cancer, kidney cancer, bladder cancer, head and neck cancers, and various hematological malignancies [98]. Immunotherapies can be combined with additional treatments such as chemotherapy, radiation therapies, or other immunotherapies to augment therapeutic efficacy and surmount resistance. Therefore, immunotherapy is advantageous over traditional therapy, providing standard care for patients with durable responses, improved quality of life, fewer side effects, and new avenues for cancer treatment.

## 6. Challenges of Immunotherapy

Despite immunotherapy having established a great impact on cancer treatment, offering long-lasting clinical benefit to some patients, it still faces several significant challenges in clinical outcomes. One of the most challenging barriers is the complexity and heterogeneity of tumors, making it harder to recognize a specific antigen to be targeted by immunotherapy, which may lead to immune evasion [99]. There are various challenges in the successful accomplishment of immunotherapy, such as the fact that all patients respond in a different way to immunotherapy, making it difficult to predict patients’ responses and to deliver consistent efficacy [100]. To predict the patient’s response to immunotherapies, definitive biomarkers are lacking for successful treatment [101]. The development of resistance is another major hurdle in the application of immunotherapy; tumor cells can modulate the TME, downregulate the antigen expression, or secrete factors that lead to immunosuppression [102]. Other challenges involve high treatment costs, limitations of an accurate animal model that replicates the effect of immunotherapy in humans and limited availability of resources [101]. To overcome these challenges, further development is crucial for more effective clinical application (Table 10).

## 7. Future Perspectives

**Figure 12:**
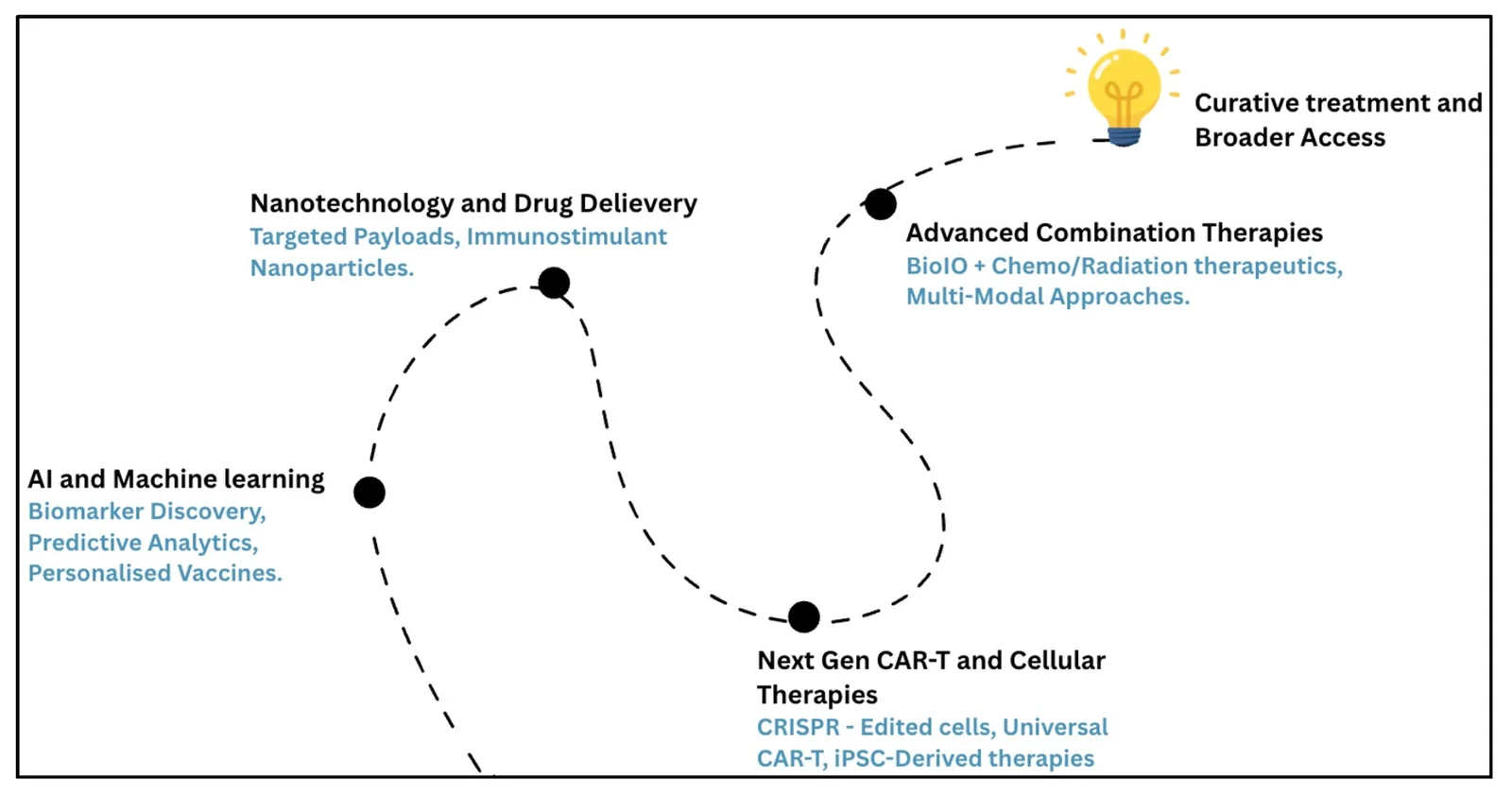
Future roadmap of cancer immunotherapy.

Immunotherapy has evolved from an experimental method to clinical implementation for cancer treatment with an increasing rate of FDA approvals. However, various challenges lead to the necessity for future research to advance the existing cancer immunotherapies and provide better clinical outcomes. Personalized (PM) medicine or precision medicine and a nanotechnology-based delivery system are proposed to be crucial for future developments by augmenting treatment efficacy and accessibility [107]. PM is an emerging cancer treatment approach that aims to tailor immunotherapy based on patients’ specific genetic, immune profiles and lifestyle factors. With the advent of next-generation immunotherapies, future directions aim to develop more effective CAR-T cells for effectiveness in solid tumors, enhanced specificity, persistence and reduced toxicity [108]. Integration of artificial intelligence (AI) into cancer treatment is also a pivotal future perspective, as AI can offer enhanced prediction of patient response, personalized and effective treatment and discovering novel therapeutic targets like neoantigens [109] (Table 11). Combination strategies have immense potential in addressing the challenges and improving therapeutic efficiency in cancer treatment, making it a promising approach for future development [110]. Hence, future development will likely focus on overcoming the ongoing challenges in cancer immunotherapy, achieving better clinical outcomes and reducing toxicity (Figure 12).

## 8. Conclusions

**Figure 13:**
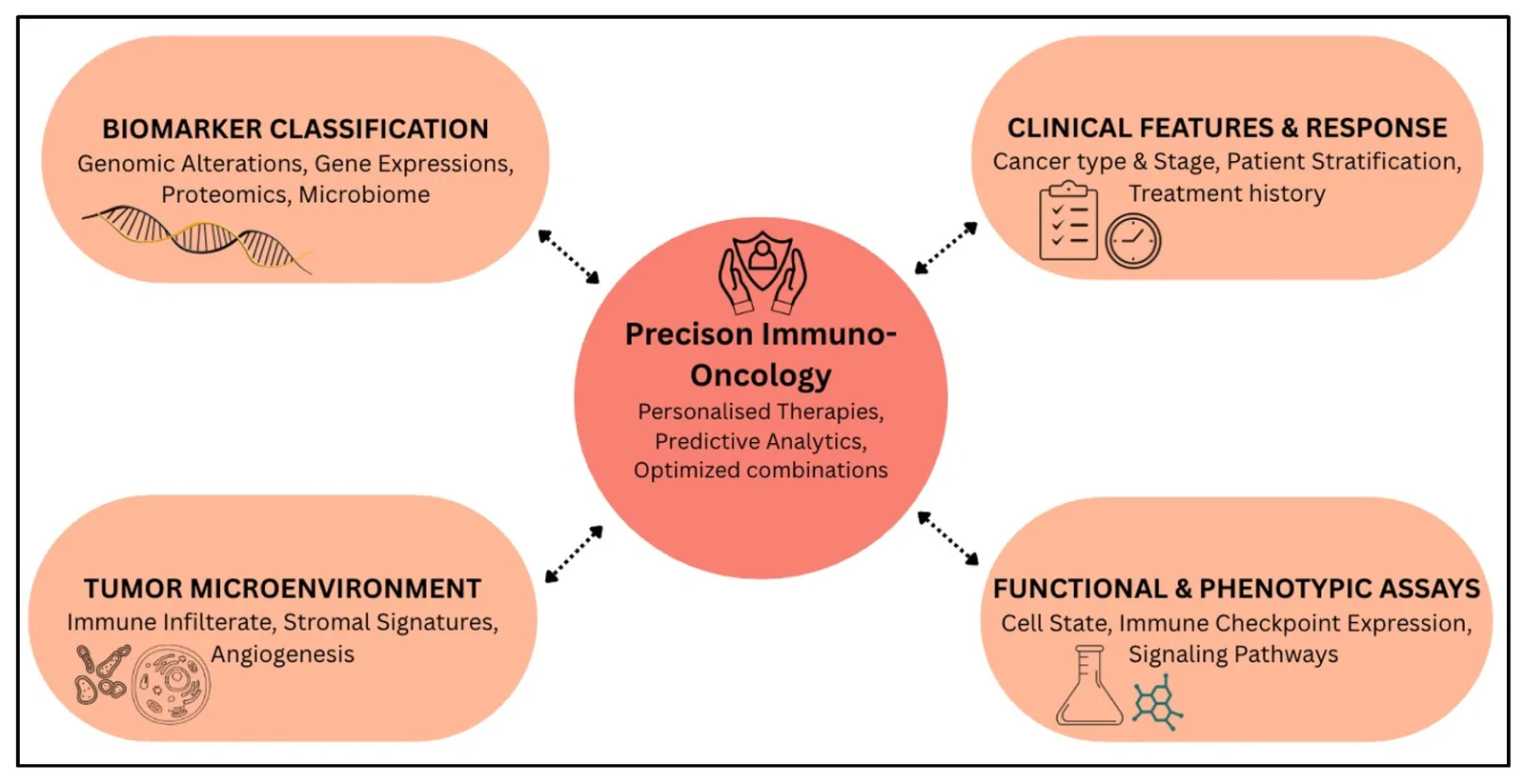
Integrative diagram illustrating the convergence of genomic, immune, and tumor microenvironment classification frameworks into precision immuno-oncology.

By using the body’s own immune system to combat cancer, immunotherapy has revolutionized the field and achieved a significant milestone in cancer treatment. Cancer immunotherapies have advantages over traditional methods like surgery, chemotherapy and radiotherapy, by offering long-term remission, fewer side effects and targeted treatment. Practice of immunotherapy, especially in combination strategies, has expanded rapidly in cancer therapeutics while providing benefits across a wide range of malignancies (Figure 13). Though it represents one of the promising approaches in standard cancer-cure and is a hope for many cancer patients, it still faces some challenges in clinical applications. To overcome the current persisting obstacles in cancer immunotherapies, future perspectives focus on improving the effectiveness of immunotherapy through personalized, next-generation, AI-based and combination strategies. The continued innovations and expanding immunotherapy research will lead to more effective and accessible approaches in cancer treatment globally.

## Figures and Tables

**Table 1:** Classification of immunotherapies with FDA-approved examples.

Category	Sub-Type	Examples	Key Mechanism
Immune checkpoint Inhibitors	PD-1 inhibitors	Nivolumab, Pembrolizumab	To restore T-cell-mediated anti-tumor immunity, block the PD-1 receptor.
	PD-L1 inhibitors	Atezolizumab, Durvalumab, Avelumab	Prevent PD-L1 binding to PD-1, restoring T-cell activation
	CTLA-4 inhibitors	Ipilimumab	Inhibit CTLA-4 to enhance T-cell activation and proliferation
Adoptive Cell Therapy	CAR-T cell therapy	Tisagenlecleucel, Axicabtagene ciloleucel	Genetically engineered T cells targeting tumor-specific antigens
	Tumor-infiltrating lymphocytes (TILs)	Lifileucel	Patient-derived tumor-reactive T cell expansion and reinfusion
	T-cell receptor (TCR) therapy	Afamitresgene autoleucel	TCR-engineered T cells targeting intracellular tumor antigens
Cancer Vaccines	Peptide/DNA vaccines	VGX-3100	Induce antigen-specific immune activation
	Therapeutic vaccines	Sipuleucel-T	Stimulate immune response against tumor-associated antigens
Monoclonal Antibodies	Tumor-targeting mAbs	Trastuzumab, Rituximab, Cetuximab	Bind tumor-associated antigens, resulting immune-mediated cytotoxicity
	Antibody–drug conjugates (ADCs)	Brentuximab vedotin, T-DM1	Use antibodies to deliver cytotoxic agents directly to cancer cells.
Cytokine Therapy	Interleukins	Aldesleukin (IL-2)	Enhance proliferation and activation of immune cells
	Interferons	IFN-α	Increase antigen presentation and immune activation
Oncolytic Virus Therapy	Genetically modified viruses	Talimogene laherparepvec (T-VEC)	Activate immunity while selectively infecting and lysing tumor cells
Bispecific Antibodies	T-cell engagers	Blinatumomab	Redirect T cells to tumor cells by dual antigen binding
Immune Modulators	Toll-like receptor agonists	Imiquimod	Use TLR signaling to initiate the innate immune response

**Table 2:** FDA-approved immune checkpoint inhibitors.

Drug Name	Target	Year of FDA Approval	Indication(s)	Key Outcomes
Ipilimumab	CTLA-4	2011	Metastatic melanoma	First ICI to show overall survival (OS) benefit in melanoma
Nivolumab	PD-1	2014	Melanoma, NSCLC, RCC, Hodgkin lymphoma, HNSCC, urothelial carcinoma, CRC (MSI-H)	Improved OS and durable responses across multiple malignancies
Pembrolizumab	PD1	2014	Melanoma, NSCLC, HNSCC, urothelial carcinoma, gastric cancer, CRC (MSI-H), TNBC	First tissue-agnostic approval for MSI-H/dMMR tumors; improved OS and PFS
Atezolizumab	PD-L1	2016	Urothelial carcinoma, NSCLC, TNBC, HCC	PD-L1-positive cancers with higher response and survival rates
Durvalumab	PD-L1	2017	NSCLC, urothelial carcinoma,	Consolidation therapy significantly improves OS and PFS in stage III NSCLC.
Avelumab	PD-L1	2017	Merkel cell carcinoma, urothelial carcinoma	Durable responses, particularly in rare aggressive cancers
Cemiplimab	PD-1	2018	Cutaneous squamous cell carcinoma, NSCLC	High response rates in advanced cutaneous SCC
Dostarlimab	PD-1	2021	Endometrial cancer	Strong and persistent responses in mismatch repair–deficient tumors
Relatlimab (with nivolumab)	LAG-3	2022	Unresectable or metastatic melanoma	First LAG-3 inhibitor; improved PFS compared to nivolumab alone

**Table 3:** Adoptive cell therapy types and approvals.

Therapy	Target/Antigen	Target Cancer Type	Clinical Development Status	Challenges
CAR-T cell therapy (Tisagenlecleucel)	CD19	B-cell acute lymphoblastic leukemia (B-ALL), DLBCL	Approved	Cytokine release syndrome (CRS), neurotoxicity, high expense
CAR-T cell therapy (Axicabtagene ciloleucel)	CD19	DLBCL, primary mediastinal B-cell lymphoma	Approved	CRS, immune effector cell-associated neurotoxicity (ICANS)
CAR-T cell therapy (Brexucabtagene autoleucel)	CD19	Mantle cell lymphoma, B-ALL	Approved	Severe toxicity, manufacturing complexity
CAR-T cell therapy (Idecabtagene vicleucel)	BCMA	Multiple myeloma	Approved	Relapse due to antigen loss and limited persistence
Tumor-infiltrating lymphocytes (TILs)	Multiple tumor antigens	Metastatic melanoma	Approved	Requires tumor resection, limited efficacy outside melanoma
TCR-engineered T cells	NY-ESO-1, MAGE-A3	Sarcoma, melanoma	Clinical trials	HLA restriction, off-target toxicity
NK cell therapy	Stress-induced ligands (e.g., NKG2D)	Leukemias, solid tumors	Clinical trials	Limited in vivo persistence, scalability issues
CIK cells	Non-MHC restricted targets	Solid and hematologic cancers	Clinical trials	Variable efficacy, lack of standardized protocols

**Table 4:** Cancer vaccine platforms.

Type	Examples	Target Cancer Type	Clinical Development Status
Preventive Vaccines	HPV vaccine (e.g., Gardasil), HBV vaccine	Cervical, anal, oropharyngeal (HPV); hepatocellular carcinoma (HBV)	FDA-approved
Therapeutic (Cell-based) vaccines	Sipuleucel-T	Prostate cancer	FDA-approved
Therapeutic (Peptide-based) vaccines	GP100 peptide vaccine	Melanoma	Clinical trials
RNA (mRNA) vaccines	mRNA-4157 (V940)	Melanoma	Clinical trials
Viral vector-based vaccines	PROSTVAC (poxvirus-based)	Prostate cancer	Clinical trials
Neoantigen vaccines	Personalized neoantigen vaccines	Melanoma, solid tumors	Clinical trials

**Table 5:** Antibody therapeutics in oncology with FDA-approved examples.

Antibody Class	Example Drug	Target	Indication
Naked monoclonal antibodies	Rituximab	CD20	Non-Hodgkin lymphoma, CLL
	Trastuzumab	HER2	HER2-positive breast and gastric cancer
	Cetuximab	EGFR	Colorectal cancer, HNSCC
	Bevacizumab	VEGF	Colorectal, lung, renal cancers
Antibody–drug conjugates (ADCs)	Brentuximab vedotin	CD30	Hodgkin lymphoma
	Trastuzumab emtansine (T-DM1)	HER2	HER2-positive breast cancer
	Trastuzumab deruxtecan	HER2	Breast, gastric cancer
Bispecific antibodies	Blinatumomab	CD19/CD3	B-cell ALL
	Amivantamab	EGFR/MET	NSCLC
Radioimmunotherapy antibodies	Ibritumomab tiuxetan	CD20	Non-Hodgkin lymphoma

**Table 6:** Strategies for Tumor Microenvironment Modulation.

Target		Intervention Type	Mechanism	Clinical Development Status
Immunosuppressive Cell Targeting	Regulatory T cells (Tregs)	Anti-CD25 antibodies	Functional inhibition or reduction of immunosuppressive Tregs	Phase I/II
	Myeloid-derived suppressor cells (MDSCs)	Small-molecule inhibitors	Reduce the accumulation of immunosuppressive myeloid cells	Phase I/II
	Tumor-associated macrophages (TAMs)	CSF-1R inhibitors	Reprogram or deplete M2-like macrophages	Phase I/II
TME Structural Remodeling	Angiogenesis (VEGF)	Anti-VEGF antibodies/TKIs	Enhance immune infiltration and restore normal tumor vasculature.	Approved
	Cancer-associated fibroblasts (CAFs)	FAP-targeted therapies	Reduce stromal immunosuppression and fibrosis	Preclinical/Phase I
	Hypoxia	Hypoxia-activated prodrugs	Target hypoxic tumor regions to enhance immune response	Phase II
Immunoregulatory Signaling Modulation	Cytokine milieu	Cytokine blockade or supplementation	Modulate pro- and anti-inflammatory signaling	Phase I/II
	Metabolic pathways (IDO, adenosine)	Enzyme inhibitors	Relieve metabolic suppression of T cells	Phase I/II

**Table 7:** Approved and emerging cytokine therapies.

Cytokine	Mechanism	Target Cancer Type	Clinical Development Status	Limitations
Interleukin-2 (IL-2)	Stimulates T cells and NK cells proliferation and activation	Metastatic melanoma, RCC	Approved	Severe systemic toxicity, vascular leak syndrome
Interferon-α (IFN-α)	Improves immune activation and antigen presentation	Melanoma, hematologic malignancies	Approved	Flu-like symptoms, fatigue, limited efficacy
Interleukin-15 (IL-15)	Promotes NK and CD8^+^ T-cell survival without Treg expansion	Leukemia, solid tumors	Phase I/II	Short half-life, immune-related toxicity
Interleukin-12 (IL-12)	Induces Th1 immune responses and IFN-γ production	Melanoma, ovarian cancer	Phase I/II	Systemic inflammation, dose-limiting toxicity
Interleukin-21 (IL-21)	Enhances cytotoxic T and NK cell function	Melanoma, lymphoma	Phase I/II	Limited clinical benefit as monotherapy
GM-CSF	Stimulates dendritic cell and macrophage maturation	Melanoma, prostate cancer (vaccine adjuvant)	Phase II/adjunct use	Variable efficacy, inflammatory side effects
TNF-α (regional therapy)	Direct tumor cytotoxicity and vascular disruption	Soft tissue sarcoma (isolated limb perfusion)	Approved (restricted use)	Severe systemic toxicity limits systemic use
Pegylated IL-2 variants	Selective immune activation and enhanced pharmacokinetics	Solid tumors	Phase II/III	Still associated with immune-related toxicity

**Table 8:** Oncolytic viruses in clinical application.

Virus	Modifications	Target Cancer Type	Clinical Development Status
Talimogene laherparepvec (T-VEC)	HSV-1 engineered to express GM-CSF; deletion of ICP34.5 and ICP47	Melanoma	FDA approved
Pexastimogene devacirepvec (Pexa-Vec/JX-594)	GM-CSF-expressing vaccine virus; deletion of thymidine kinase	Hepatocellular carcinoma	Phase III
Oncorine (H101)	Adenovirus with E1B-55kDa deletion	Head and neck cancer	Approved in China; Phase II globally
DNX-2401	RGD-modified conditionally replicating adenovirus	Glioblastoma	Phase II
CG0070	Adenoviruses that express GM-CSF and selective replication via the RB pathway	Bladder cancer	Phase II/III
Reolysin (Pelareorep)	Wild-type reovirus (RAS-selective replication)	Breast, pancreatic, head and neck cancers	Phase II/III
HF10	Naturally attenuated HSV-1	Melanoma, pancreatic cancer	Phase II
VSV-IFNβ	Vesicular stomatitis virus expressing interferon-β	Solid tumors	Phase I/II
PVSRIPO	Recombinant poliovirus–rhinovirus chimera	Glioblastoma	Phase II

**Table 9:** Clinical use of immunotherapy by cancer type.

Target Cancer Type	Therapy	FDA-Approved Drugs	Survival/Response Outcomes
Melanoma	Immune checkpoint inhibitors (ICI)	Ipilimumab, Nivolumab, Pembrolizumab, Relatlimab	Significant improvement in overall survival with durable long-term responses [89]
Renal cell carcinoma (RCC)	ICIs and ICI–TKI combinations	Nivolumab, Pembrolizumab, Ipilimumab	Durable responses and OS benefit in advanced disease [90]
Hodgkin lymphoma	PD-1 inhibitors	Nivolumab, Pembrolizumab	High overall response rates in relapsed/refractory disease [91]
Triple-negative breast cancer (TNBC)	ICIs + chemotherapy	Pembrolizumab, Atezolizumab	Improved PFS and OS in PD-L1-positive tumors [92]
Hepatocellular carcinoma (HCC)	ICIs ± anti-angiogenics	Nivolumab, Pembrolizumab, Atezolizumab	Improved survival in advanced HCC [93]
Prostate cancer	Cancer vaccine	Sipuleucel-T	Modest OS benefit with minimal effect on tumor burden [94]

**Table 10:** Key limitations & strategies to overcome.

Challenge	Underlying Cause	Mitigation Strategies
Primary or acquired resistance	Loss of antigen presentation, reduced tumor mutational burden, and tumor immune evasion	Combination therapies (ICI + chemotherapy/radiotherapy), novel checkpoints (LAG-3, TIM-3), biomarker-guided patient selection [103]
Tumor heterogeneity	Inter- and intra-tumoral genetic and phenotypic variability	Personalized immunotherapy, multi-antigen targeting, combination immunotherapeutic approaches [104]
Immunosuppressive tumor microenvironment (TME)	Tregs, MDSCs, and inhibitory cytokines (TGF-β, IL-10) are present.	TME-modulating agents, TGF-β inhibitors, depletion of suppressive immune cells [56]
Lack of reliable predictive biomarkers	Incomplete understanding of immune response dynamics	Development of composite biomarkers (PD-L1 + TMB + gene signatures), liquid biopsies [105]
Short durability of response	Adaptive resistance mechanisms and immune fatigue	Maintenance immunotherapy, sequential treatment strategies, immune memory enhancement [106]

**Table 11:** Next-generation platforms under development.

Modality	Example	Potential Advantages	Clinical Development Status
Bispecific T-cell engagers (BiTEs)	Blinatumomab, CD3×CD20 bispecific	MHC-independent action and direct T-cell rerouting to tumor cells	FDA-approved (blinatumomab); others in Phase I–III [111]
CAR-T cell therapy (next-gen)	Dual-target CAR-T (CD19/CD22), armored CAR-T	Reduced antigen escape, improved persistence and efficacy	Phase I–II [112]
Personalized neoantigen vaccines	mRNA-based neoantigen vaccines	High tumor specificity, minimal off-target toxicity	Phase I–II [113]
Oncolytic virus therapy	T-VEC, adenovirus-based vectors	Selective tumor lysis, immune priming	FDA-approved (T-VEC); others in clinical trials [114]
Immune checkpoint combinations	PD-1 + LAG-3, PD-1 + TIGIT inhibitors	Overcome resistance, enhanced anti-tumor immunity	Phase II–III [115]
TCR-engineered T cells	NY-ESO-1-specific TCR therapy	Target intracellular antigens, high specificity	Phase I–II [116]
Nanoparticle-based immunotherapy	Lipid nanoparticles delivering mRNA or antigens	Targeted delivery, reduced systemic toxicity	Preclinical–Phase II [117]

## Data Availability

Additional data will be provided upon request.
